# Development of dual SST_2_/SST_5_ targeting radioligands through rational structural design

**DOI:** 10.1039/d6md00646a

**Published:** 2026-10-05

**Authors:** Fabienne Spinnler, Luigi Del Pozzo, Lennart Bohrmann, Sandra Zanger, Nina Kathleen Blum, Monique Brendel, Stefan Schulz, Rosalba Mansi, Melpomeni Fani

**Affiliations:** a Division of Radiopharmaceutical Chemistry, Department Theragnostics, University Hospital Basel, University of Basel Basel 4031 Switzerland rosalba.mansi@usb.ch melpomeni.fani@usb.ch +41(0)61 556 58 91; b Institute of Pharmacology and Toxicology, University Hospital Jena, Friedrich Schiller University Jena 07747 Germany

## Abstract

Radioligand therapy targeting somatostatin receptors has emerged as a highly effective treatment strategy for neuroendocrine tumors, with growing evidence indicating that SST_2_ antagonists and dual SST_2_/SST_5_ targeting approaches may further enhance tumor coverage and therapeutic efficacy. In this study, DOTA-NOC, a somatostatin analog with high affinity for SST_2_ and SST_5_, served as the scaffold for the design of novel ligands incorporating structural features associated with receptor antagonism. The resulting ligands were conjugated to DOTA for ^177^Lu radiolabeling and evaluated for receptor affinity, antagonistic potency, and *in vivo* properties. Among the series, ^177^Lu-10 displayed high affinity for both receptor subtypes while acting selectively as an SST_5_ antagonist. Compared to [^177^Lu]Lu-DOTA-NOC, it showed similar SST_2_ tumor uptake but nearly 5-fold higher accumulation in SST_5_-expressing tumors. This work provides new insights into the structural determinants underlying SST_2_ and SST_5_ affinity, selectivity, and antagonistic behavior, demonstrating that receptor recognition and functional activity arise from the combined contribution of multiple structural elements and supporting the development of dual SST_2_/SST_5_-targeted radioligands.

## Abstract 2

Development of a high affinity dual SST_2_ agonist/SST_5_ antagonist radioligand for improved targeting and imaging of somatostatin receptor-expressing tumors.

## Graphical abstract

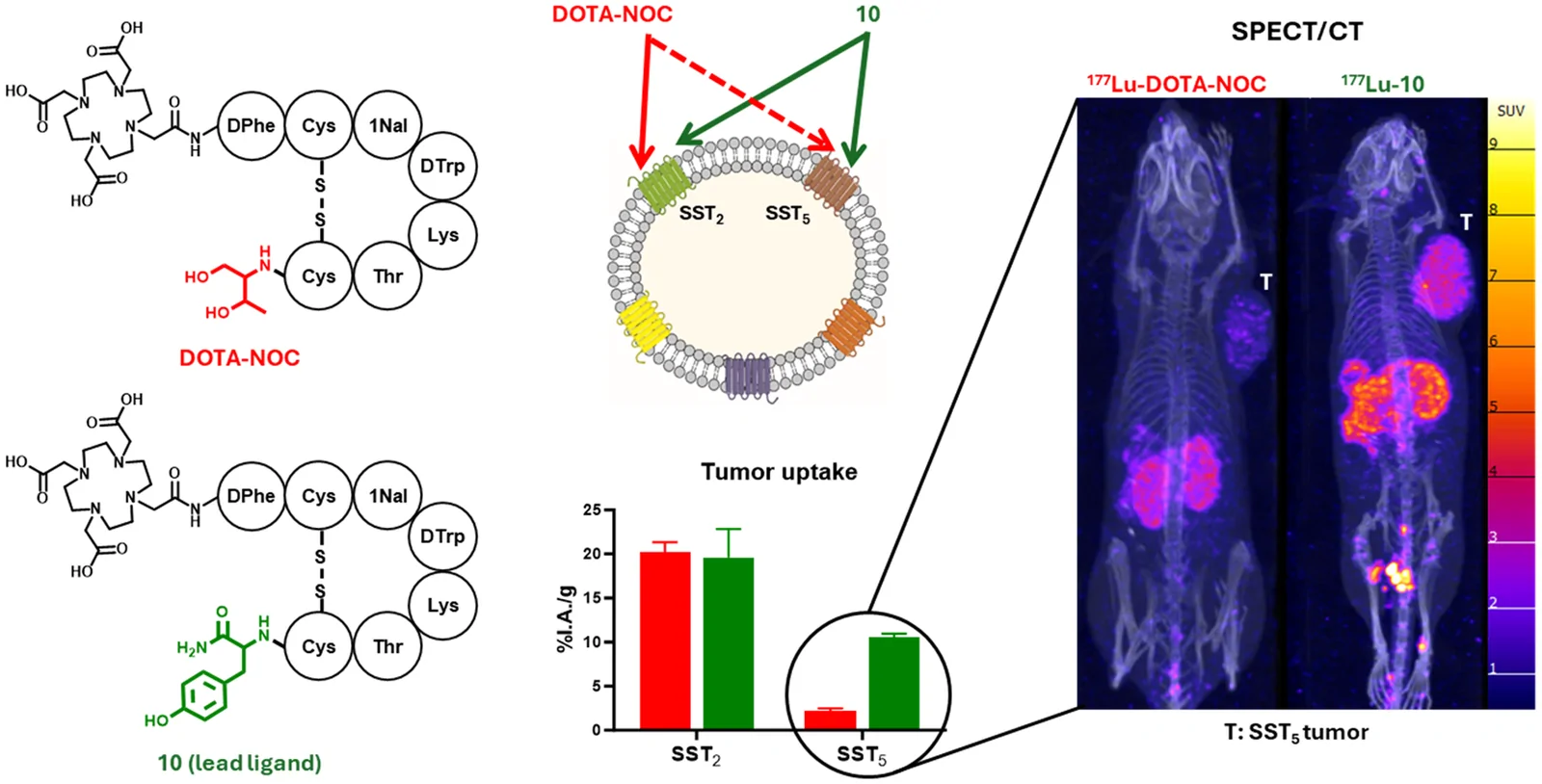

## 1. Introduction

Targeted radioligand therapy using radiolabeled somatostatin analogs has proven highly effective for treating well-differentiated, inoperable, and metastatic neuroendocrine tumors (NETs), particularly those of gastro-enteropancreatic (GEP) origin. The two key radioligands are [^177^Lu]Lu-DOTA-TATE (Lutathera®, Novartis) and [^177^Lu]Lu-DOTA-TOC (currently completed phase III COMPETE trial, NCT03049189).^1–3^ The success of these therapies is largely due to the high expression of somatostatin receptor subtype 2 (SST_2_) in NETs, which serves as the primary molecular target for both radioligands. Structurally, DOTA-TATE and DOTA-TOC (DOTA-dPhe-c(Cys-Tyr-dTrp-Lys-Thr-Cys)-Thr-OH and -Thr(ol), respectively) are very similar. They are cyclic octapeptides containing a disulfide-bridged six-membered ring (derivatives of octreotide)^4^ conjugated at the N-terminus with the chelator DOTA (1,4,7,10-tetraazacyclododecane-1,4,7,10-tetraacetic acid), enabling radiolabeling with lutetium-177. The sole difference between the two molecules lies at the C-terminus: DOTA-TATE features a threonine (Thr-OH), while DOTA-TOC has a threoninol (Thr(ol)). This subtle variation imparts DOTA-TATE with markedly higher SST_2_ selectivity and approximately 10-fold greater affinity (IC_50_ ∼1.5 nM *vs.* ∼14 nM for DOTA-TOC).^5^

Emerging preclinical and clinical evidence suggests that radiolabeled SST_2_ antagonists may provide superior therapeutic performance in comparison to the agonists DOTA-TATE and DOTA-TOC.^6–9^ In contrast to agonists, which rely on receptor activation and internalization, SST_2_ antagonists bind to a greater number of receptors, leading to higher tumor accumulation and prolonged tumor residence time, ultimately resulting in increased absorbed radiation dose.^10–13^ Both classes of radiolabeled somatostatin-based ligands share the general structure of the octapeptide with a six-member ring but differ mainly in the inverted chirality in positions 1 and 2 (d^1^/l^2^ for the agonists and l^1^/d^2^ for the antagonists) and in their C-terminal residues, with antagonists containing larger hydrophobic aromatic amino acids and agonists smaller hydrophilic ones.^13,14^ Studies with the first SST_2_ antagonist DOTA-BASS (DOTA-Phe(4NO_2_)-c(dCys-Tyr-dTrp-Lys-Thr-Cys)-dTyr-NH_2_)^15^ and with the clinically used DOTA-JR11^6,16,17^ and DOTA-LM3,^18^ support the use of SST_2_ receptor antagonism as a promising strategy to enhance the efficacy of radioligand therapy, due to improved lesion detection and higher tumor radiation dose compared to SST_2_ agonists.

While advances in SST_2_-targeted radioligand therapy have been made, they do not optimally address all neuroendocrine neoplasms.^19^ Poorly differentiated neuroendocrine carcinomas (NECs), high-grade NETs, and some well-differentiated NETs often show low or heterogeneous SST2 expression,^20^ resulting in suboptimal tumor targeting with SST_2_-specific analogs.^21^ The receptor subtype 5 (SST_5_) ranks as the second most frequently expressed somatostatin receptor subtype in GEP-NETs.^22^ SST_5_ expression is reported in 38–83% of GEP-NETs, compared with 60–93% for SST_2_, based on immunohistochemical studies.^23–29^ SST_5_ expression extends beyond GEP-NETs to a variety of other tumors, including growth hormone secreting pituitary adenomas, breast cancer and intestinal or bronchial NETs,^30,31^ glioblastomas,^32^ the tumor vasculature of pancreatic ductal adenocarcinomas,^33^ certain lung cancers,^34^ and subsets of ovarian and cervical carcinomas.^35^ This positions SST_5_ as a key target together with SST_2_. These patterns support the development of dual SST_2_/SST_5_-targeted radiolabeled somatostatin analogs to broaden the spectrum of tumors amenable to radioligand therapy and enhance efficacy by increasing the radiation dose delivery to tumors co-expressing both subtypes.

To enable dual SST_2_/SST_5_ targeting, while leveraging the advantages seen with SST_2_ antagonists, we pursued an approach that aims to incorporate both properties in a single ligand. We began our ligand development with the well-known SST_2_ agonist DOTA-NOC (DOTA-dPhe-c(Cys-1Nal-dTrp-Lys-Thr-Cys)-Thr(ol)),^36^ which also exhibits substantial SST_5_ affinity, owing to the 1-naphthylalanine residue at position 3 instead of tyrosine.^36,37^ We investigated whether DOTA-NOC could be converted into an SST_2_/SST_5_ antagonist by introducing structural motifs characteristic of SST_2_-selective antagonists,^13,15,38,39^ while maintaining its dual targeting. Sixteen novel ligands were designed, synthesized and radiolabeled with ^177^Lu. The newly generated radioligands were evaluated *in vitro* and *in vivo* for SST_2_/SST_5_ dual-targeting and antagonism. DOTA-NOC was used in all studies as reference.

## 2. Results and discussion

### 2.1 Design and affinity data of DOTA-NOC-based ligands

Using DOTA-NOC as the starting scaffold (Fig. 1), we investigated the impact of: i) inversion of chirality at positions 1 and 2; ii) incorporation of an aromatic amino acid at the C-terminus;^13^ iii) inverted chirality combined with substituted phenylalanines at position 1; and iv) combinations thereof. Lastly, we explored the effect of Val in position 6, as it is known to enhance SST_5_ affinity in other octapeptide somatostatin analogs, like DOTA-LAN (LAN = lanreotide)^40^ and DOTA-VAN (VAN = vapreotide).^41^

**Fig. 1:**
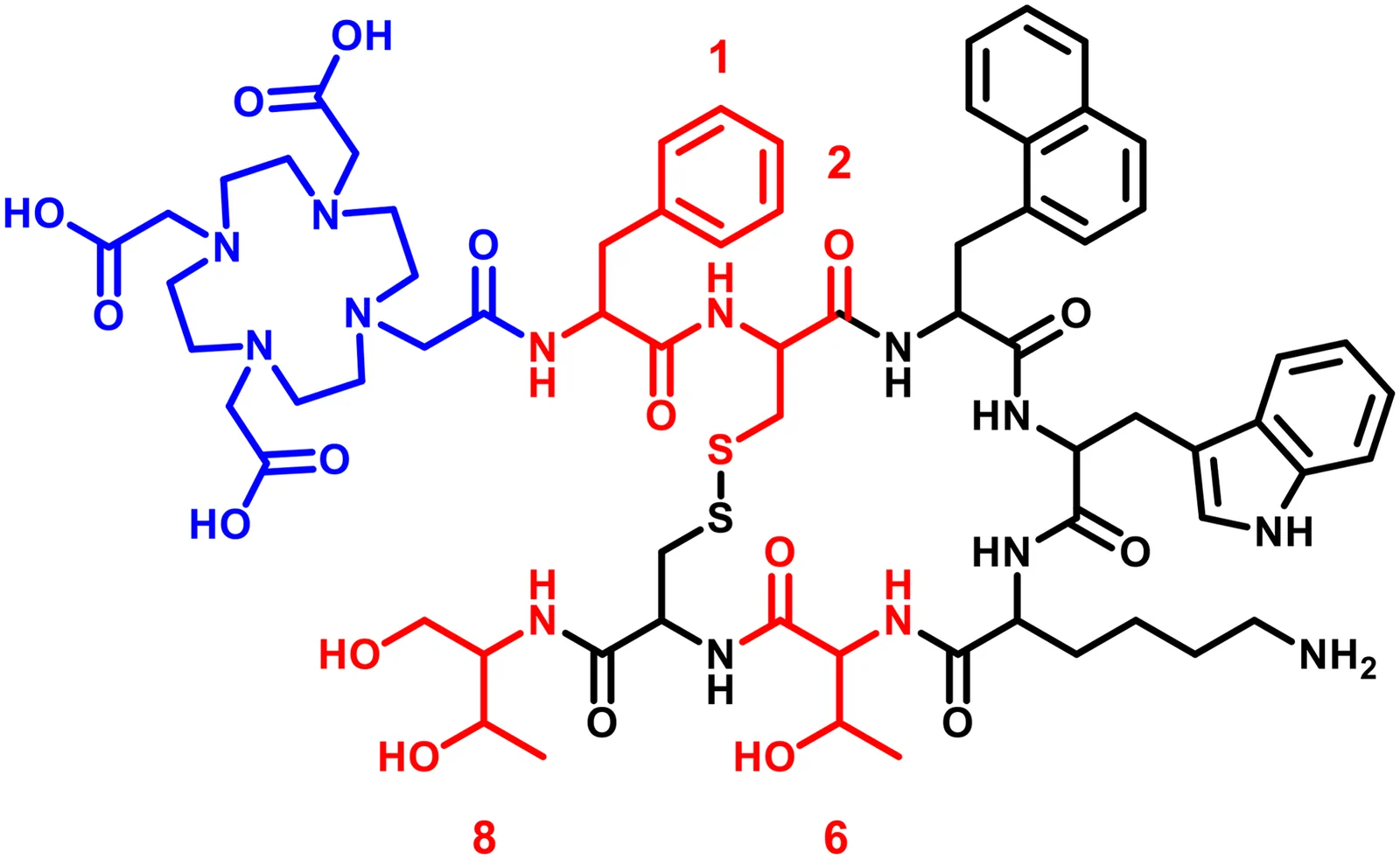
Structure of DOTA-NOC; blue: DOTA chelator, red: positions 1, 2, 6 and 8 modified in this study.

All DOTA-conjugated peptide derivatives of DOTA-NOC (hereafter referred to as ligands) are presented in Table 1, with the respective modifications highlighted in color, together with the half-inhibition concentration (IC_50_) determined by competition binding with [^125^I]-Tyr-SS-14 (SS-14 = somatostatin-14) on HEK-SST_2_ and CHO-SST_5_ membranes.

**Table 1:** Peptide sequences of ligands under investigation designed based on the reference DOTA-NOC and their binding affinity data, expressed as half-inhibition concentration (IC_50_) in competition with [^125^I]-Tyr-SS-14 on HEK-SST_2_ and CHO-SST_5_ membranes (at least two individual experiments, each in triplicate). CI = confidence of interval. Summary of 7TM phosphorylation assay results (*n* = 4): characterization of ligands based on their ability to inhibit SS-14-induced phosphorylation of pS341/pS343-SST_2_ and pT333-SST_5_

	Code	Peptide sequence	IC_50_ (nM (95% CI))		Antagonism	
			SST_2_	SST_5_	SST_2_	SST_5_
	**DOTA-NOC**	DOTA-dPhe-c[Cys-1Nal-dTrp-Lys-Thr-Cys]-Thr(ol)	0.36 (0.16–0.39)	13.8 (12.2–15.6)	No	Yes
Series 1	1	DOTA-**Phe**-c[**dCys**-1Nal-dTrp-Lys-Thr-Cys]-Thr(ol)	2.6 (2.0–3.2)	>1000	No	—
	2	DOTA-**dPhe**-c[**dCys**-1Nal-dTrp-Lys-Thr-Cys]-Thr(ol)	47.3 (40.2–55.8)	334 (273–407)	—	—
	3	DOTA-**Phe**-c[**Cys**-1Nal-dTrp-Lys-Thr-Cys]-Thr(ol)	0.61 (0.47–0.78)	49 (36–67)	No	Yes
	4	DOTA-**Phe(4NO_2_)**-c[**dCys**-1Nal-dTrp-Lys-Thr-Cys]-Thr(ol)	1.4 (1.2–1.7)	294 (229–379)	No	No
	5	DOTA-**Phe(4NO_2_)**-c[**dCys**-1Nal-dTrp-Lys-**Val**-Cys]-Thr(ol)	4.8 (2.4–8.9)	>1000	No	—

Series 2	6	DOTA-Phe-c[dCys-1Nal-dTrp-Lys-Thr-Cys]-**Thr-NH_2_**	6.0 (4.3–8.4)	>1000	—	—
	7	DOTA-Phe-c[dCys-1Nal-dTrp-Lys-Thr-Cys]-**Thr-OH**	5.3 (3.4–8.1)	>1000	—	—
	8	DOTA-**Phe(4Cl)**-c[dCys-1Nal-dTrp-Lys-Thr-Cys]-Thr-OH	1.3 (1.2–1.4)	>1000	—	—
	9	DOTA-**Phe(4NO_2_)**-c[dCys-1Nal-dTrp-Lys-**Val**-Cys]-Thr-OH	2.4 (1.6–3.6)	>1000	—	—

Series 3	10	DOTA-dPhe-c[Cys-1Nal-dTrp-Lys-Thr-Cys]-**dTyr-NH_2_**	0.56 (0.44–0.70)	3.6 (2.9–4.4)	No	Yes
	11	DOTA-**Phe**-c[**dCys**-1Nal-dTrp-Lys-Thr-Cys]-dTyr-NH_2_	4.5 (3.9–5.2)	>1000	Yes	—
	12	DOTA-**Phe(4NO_2_)**-c[dCys-1Nal-dTrp-Lys-Thr-Cys]-dTyr-NH_2_	6.0 (5.5–6.6)	449 (323–621)	Yes	No
	13	DOTA-**Phe(4NO_2_)**-c[dCys-1Nal-dTrp-Lys-**Val**-Cys]-dTyr-NH_2_	9.1 (5.5–15.4)	784 (636–975)	Yes	No
	14	DOTA-**Phe(4NO_2_)**-c[dCys-1Nal-dTrp-Lys-Thr-Cys]-**Phe(4Cl)-OH**	2.9 (2.2–3.9)	>1000	Yes^a^	—
	15	DOTA-**Phe(4Cl)**-c[dCys-1Nal-dTrp-Lys-Thr-Cys]-Phe(4Cl)-OH	2.4 (1.9–3.1)	>1000	—	—
	16	DOTA-**Phe(4Cl)**-c[dCys-1Nal-dTrp-Lys-Thr-Cys]-**2Nal-NH_2_**	55.4 (20.6–375)	41 (30–57)	—	—

a Weak antagonistic effect (100 μM); — not done as the ligands had no affinity for SST_5_ and/or SST_2_.

All the ligands were obtained with >95% purity, based on RP-HPLC analysis. The analytical data are provided in Table S1 and Fig. S1. The designed ligands are grouped in three series, based on their C-terminus:

**Series 1** (1–5), in which Thr(ol)^8^ at the C-terminus is retained, while altering the chirality at positions 1 and 2. In relation to DOTA-NOC (chirality d^1^/l^2^), ligands 1–3 include the different combinations in these two positions. Focusing on the inverted chirality (l^1^/d^2^) known from SST_2_ antagonists, the substituted Phe(4NO_2_)^1^ was inserted in position 1 (4) and in addition, Val^6^ in position 6 (5).

**Series 2** (6–9), in which the C-terminus was modified to either threonine amide (Thr^8^-NH_2_, 6) to increase stability,^42^ or to threonine hydroxyl (Thr^8^-OH, 7) mimicking known modification between DOTA-NOC and DOTA-NOC-ATE or DOTA-TOC and DOTA-TATE.^43^ In all cases, the inverted chirality in positions 1 and 2 was preserved. Furthermore, Phe(4Cl)^1^ was introduced in position 1 (8) and in addition Phe(4NO_2_)^1^ in combination with Val^6^ in position 6 (9).

**Series 3** (10–16), in which the C-terminus was substituted by aromatic and/or hydrophobic amino acids. Initially, dTyr^8^-NH_2_ was introduced at the C-terminus (ligands 10–13), a modification reported to improve hydrophilicity^38^ without or in combination with inverted chirality (d^1^/l^2^, 10 and l^1^/d^2^, 11, respectively). By preserving the inverted chirality, the dTyr^8^-NH_2_-series was completed with the introduction of Phe(4NO_2_)^1^ (12), and the combination of Phe(4NO_2_)^1^ and Val^6^ (13). Alternatively, the C-terminus incorporated the aromatic residue Phe(4Cl) in combination with substituted Phe(4NO_2_)^1^ (14) or Phe(4Cl)^1^ (15). Lastly, the sterically demanding amino acid 2Nal-NH_2_ was used at the C-terminus, as previous studies by Hocart *et al.*^39,44^ suggested, in combination with Phe(4Cl)^1^ (16).

All the ligands exhibited very high affinity for SST_2_ (IC_50_ < 10 nM), except for 2 and 16 that exhibited moderate affinity (IC_50_ ∼ 50 nM). Among the tested ligands, 3 and 10 demonstrated sub-nanomolar affinity (IC_50_ < 1 nM), similar to DOTA-NOC.

Nine out of the 16 ligands lost affinity for SST_5_, while the other 7 showed measurable affinity. Among these, 3 and 16 showed moderate affinity (IC_50_ ∼ 40–50 nM), while 10 showed the highest affinity (IC_50_ = 3.6 nM), with a 4-fold lower IC_50_ than DOTA-NOC.

### 2.2 Radiolabeling and lipophilicity (log *D*)

All the ligands were labeled with ^177^Lu in radiolabeling yield >95% and radiochemical purity >93% (Table S2, Fig. S2). The radioligands were prepared with apparent molar activities ranging from 20 to 60 MBq nmol^−1^, depending on the type of experiment performed.

The distribution coefficient (log *D*) of the ^177^Lu-labeled ligands between the organic and aqua phase ranges from −2.0 to 1.0 (Fig. 2). Numeric values are reported as mean ± standard deviation in Table S2. Compared to the reference ligand [^177^Lu]Lu-DOTA-NOC (log *D* = −1.61 ± 0.19), all the radioligands are more lipophilic, with the exception of ^177^Lu-3 and ^177^Lu-7. Three of the radioligands, ^177^Lu-13, ^177^Lu-15 and ^177^Lu-16, demonstrate high lipophilicity (log *D* > 0), a property generally considered disadvantageous for systemic *in vivo* use of targeted radioligands.

**Fig. 2:**
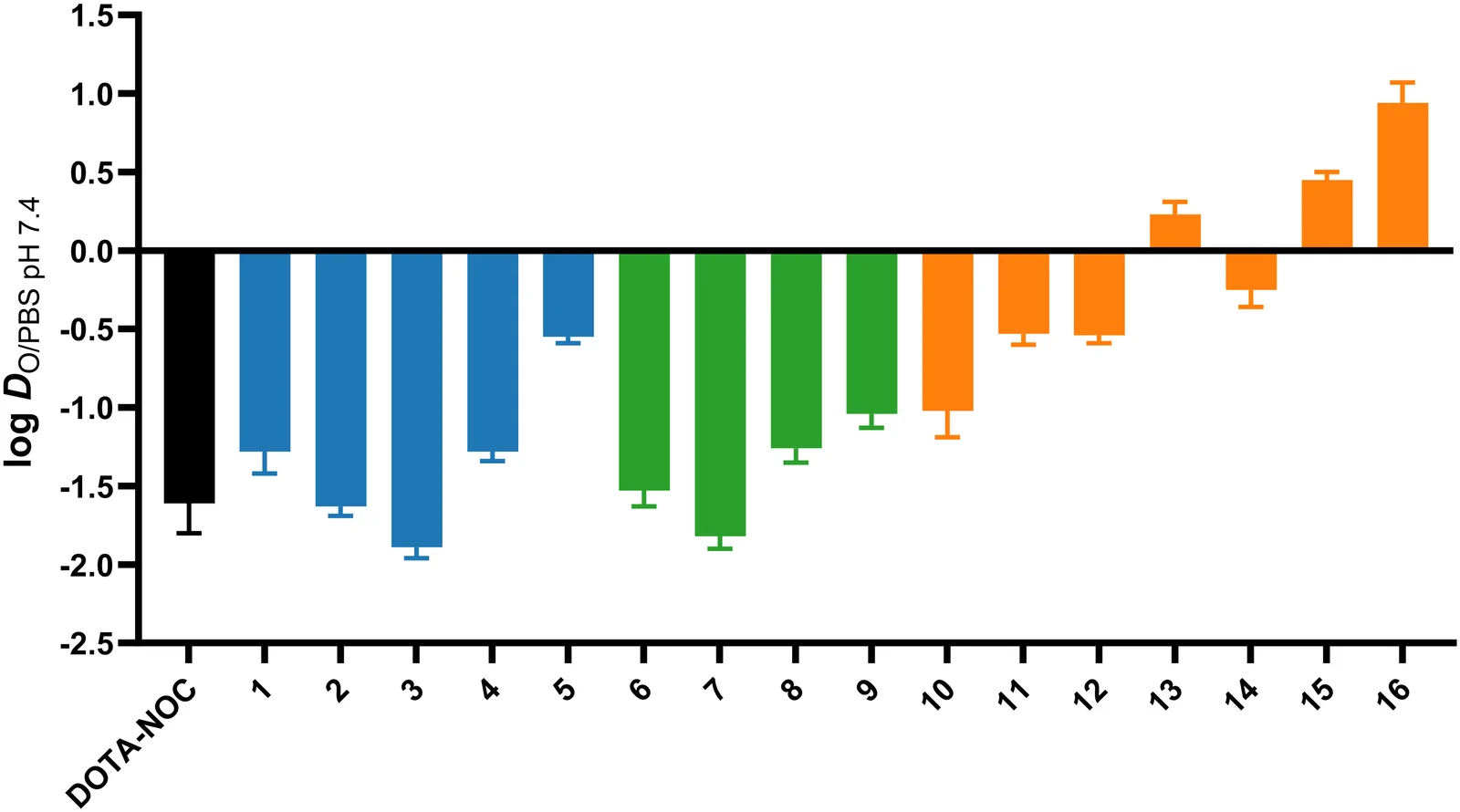
Lipophilicity of the ^177^Lu-labeled ligands was assessed by the determination of their distribution (log *D*) between octanol (O) and phosphate buffered saline (PBS) at the physiological pH of blood serum, pH = 7.4.

Lipophilic radioligands often suffer from higher nonspecific binding, higher binding to plasma proteins, and greater accumulation in off-target organs. A representative case is [^90^Y]Y-DOTA-LAN, which demonstrated modest antitumor effects and significant hepatic toxicity due to increased hepatobiliary clearance in phase I/II trials.^45,46^

### 2.3 *In vitro* evaluation

#### 2.3.1 Cellular uptake and distribution

The cellular uptake and distribution between the surface bound and internalized fraction for all ^177^Lu-labeled ligands were evaluated in HEK-SST_2_ cells at 37 °C at 1, 2 and 4 hours (Table S3). Such studies were not performed in HEK-SST_5_ cells due to the absence of ligand-induced receptor internalization of this SST subtype.^47^

The results in HEK-SST_2_ cells after 4 hours of incubation for all the radioligands are presented in Fig. 3. ^177^Lu-16 is not reported because the nonspecific uptake of this radioligand could not be reliably quantified, likely due to non-specific adhesion effects, attributable to its high lipophilicity (log *D* = 0.94 ± 0.13). Thus, 16 was excluded at this stage from further investigations.

**Fig. 3:**
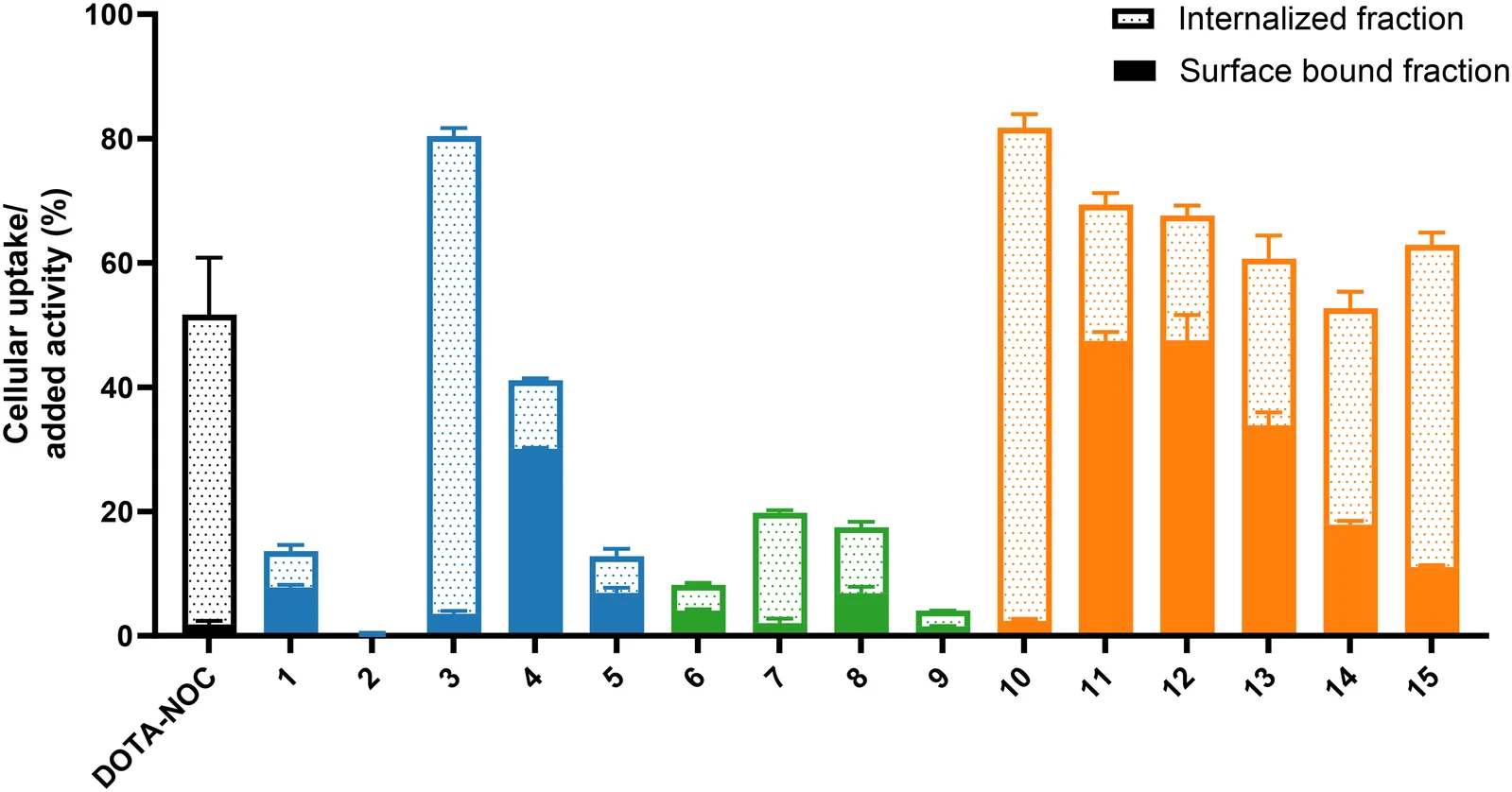
Cellular uptake and distribution of the ^177^Lu-ligands in HEK-SST_2_ cells after 4 hours of incubation at 37 °C, divided into the specific cell surface bound fraction (full bars) and internalized fraction (open colored dotted bars). The results are expressed as % (mean ± SD) of the applied activity in the cells and are normalized per million cells. All values refer to specific surface bound and internalization after subtracting the non-specific (measured in non-transfected HEK cells) from the total bound and internalized fractions.

Seven out of 15 radioligands, namely ^177^Lu-1, ^177^Lu-2, ^177^Lu-5, ^177^Lu-6, ^177^Lu-7, ^177^Lu-8 and ^177^Lu-9 were excluded from further evaluation due to their low cellular uptake (internalized fraction + surface bound fraction <20%), compared to the benchmark [^177^Lu]Lu-DOTA-NOC.

The remaining 8 radioligands showed cellular uptake >40% and can be grouped into two distinct classes, based on their distribution between the surface-bound and internalized fractions, as it is well established that radiolabeled somatostatin agonists are exclusively internalized, whereas SST_2_ antagonists predominantly remain bound to the cell membrane:^10^

I. Four radioligands, ^177^Lu-3, ^177^Lu-10, ^177^Lu-14 and ^177^Lu-15 are predominantly internalized, similar to[^177^Lu]Lu-DOTA-NOC. Notably, ^177^Lu-3 and ^177^Lu-10 demonstrated specific cellular uptake of 80% of the total added activity, which is significantly higher than [^177^Lu]Lu-DOTA-NOC (approx. 55%). The characteristics of this group are indicative of agonism in SST_2_.

II. Four radioligands, ^177^Lu-4, ^177^Lu-11, ^177^Lu-12 and ^177^Lu-13 showed a higher surface-bound fraction than internalized. The characteristics of this group are indicative of antagonism in SST_2_. Among them, ^177^Lu-11 and ^177^Lu-12 showed the highest specific cellular uptake (∼70% of the added activity), with two-thirds remaining on the cell surface.

Of the eight radioligands with cellular uptake >40%, the following six were selected for further evaluation: ^177^Lu-3, ^177^Lu-4, ^177^Lu-10, ^177^Lu-11, ^177^Lu-12 and ^177^Lu-14. The remaining two, ^177^Lu-13 and ^177^Lu-15, were excluded due to their pronounced lipophilicity (log *D* > 0, Fig. 2), and lack of any distinct advantages over the six selected radioligands.

#### 2.3.2 Saturation binding studies

Saturation binding studies were performed to determine the equilibrium dissociation constant (*K*_D_) and the maximum number of binding sites (*B*_max_) of the six selected radioligands in HEK-SST_2_ membranes. Additionally, the four ligands with measurable affinity for SST_5_ were tested in CHO-SST_5_ membranes. The results are summarized in Table S4 and illustrated in Fig. S3.

All tested radioligands exhibit very high affinity for SST_2_, with *K*_D_ values below 1.1 nM, in line with the results obtained from the inhibition assay (Table 1). Among all, ^177^Lu-3 and ^177^Lu-10 had the highest affinity with sub-nanomolar *K*_D_ comparable to [^177^Lu]Lu-DOTA-NOC.

[^177^Lu]Lu-DOTA-NOC showed the lowest *B*_max_ (0.07 nM), whereas the new radioligands exhibited 1.5- up to 3-fold higher values. This is consistent with reports of a 3- to 4-fold higher *B*_max_ for SST_2_ antagonists *vs.* SST_2_ agonists. Notably, the *B*_max_ values of ^177^Lu-11 and ^177^Lu-12 in this cell line were similar to that reported for the SST_2_ antagonist [^177^Lu]Lu-DOTA-JR11 (0.37 ± 0.02 nM),^10^ supporting an antagonistic profile. The higher *B*_max_ of SST_2_ antagonists is thought to contribute substantially to their enhanced tumor uptake and, consequently, greater tumor radiation dose in both preclinical^12,48^ and clinical settings.^7,49^ Because some radioligands showed substantial ligand depletion, saturation-binding analyses were performed using the estimated free radioligand activity to account for the reduction in ligand available for receptor binding.^50^

On SST_5_, ^177^Lu-3 and ^177^Lu-10 presented a *K*_D_ similar to [^177^Lu]Lu-DOTA-NOC, with the *B*_max_ of ^177^Lu-10 being 2-fold higher than that of [^177^Lu]Lu-DOTA-NOC (0.54 *vs.* 0.27 nM, respectively). ^177^Lu-4 and ^177^Lu-12 were tested but no saturation binding curve could be generated. Although the affinity of these two ligands was measurable in the inhibition assay, it was likely too low to generate a saturation curve under the experimental conditions used.

#### 2.3.3 Antagonistic potency

The antagonistic potency of the selected ligands, 3, 4, 10, 11, 12 and 14, was assessed and the results are reported in Table 1 and Fig. S4 and S5. Ligands 1, 5 and 13 were included to elucidate the impact of key structural modifications, as discussed below.

Inversion of chirality at positions 1 and 2 (Phe^1^/dCys^2^, 1) did not convert DOTA-NOC into an SST_2_ antagonist, contrary to previous reports for SST_2_-selective octreotide-based agonists.^15,39^ Instead, this modification led to a seven-fold reduction in SST_2_ affinity and completely abolished SST_5_ affinity. To dissect positional effects, chirality at positions 1 and 2 was varied individually. The dPhe^1^/dCys^2^ derivative of DOTA-NOC (2) showed no favorable affinity profile (Table 1), whereas the Phe^1^/Cys^2^ variant (3) improved SST_2_ affinity to levels comparable to DOTA-NOC (0.61 *vs.* 0.36 nM, respectively), and restored SST_5_ affinity. 3 retained the same functional profile in SST_2_ and SST_5_ as DOTA-NOC.

Introduction of an aromatic amino acid at the C-terminus of DOTA-NOC, like dTyr^8^-NH_2_ (10), preserved the functional profile and the high SST_2_ affinity of the scaffold (0.56 *vs.* 0.36 nM, respectively), while it improved significantly the SST_5_ affinity (3.6 *vs.* 13.8 nM, respectively). In contrast, when this modification was combined with the inverted chirality (Phe^1^/dCys^2^, dTyr^8^-NH_2_, 11) the SST_2_ affinity was reduced, and the SST_5_ affinity was abolished, consistent with the results discussed above for series 1. Notably, however, this combination displayed SST_2_ antagonism.

Introduction of a substituted l-phenylalanine, *e.g.* Phe(4NO_2_) at position 1 has previously been shown to confer SST_2_ antagonism and improve SST_2_/SST_5_ affinity.^15,38,39,44^ Here, it was incorporated either without modifying the C-terminus in series 1 (Phe(4NO_2_)^1^/dCys^2^, 4) or by introducing an aromatic C-terminal residue in series 3 (Phe(4NO_2_)^1^/dCys^2^, dTyr^8^-NH_2_, 12). In both cases, Phe(4NO_2_)^1^ enabled SST_5_ recognition (IC_50_ ∼250–450 nM), whereas the corresponding unsubstituted analogs (1 and 11) showed no SST_5_ affinity. This might indicate that the 4NO_2_ substituent contributes to SST_5_ receptor binding, likely through enhanced aromatic and polar interactions with the aromatic “hydrophobic lock” of SST_5_ and extracellular loop residues.^51–53^ These interactions might not be accessible to the unsubstituted Phe^1^ residue. Despite this shared effect, the SST_2_ functionality was different with 12 showing antagonism, while 4 did not.

Lastly, motivated by the effect of Val^6^ in the somatostatin analogs lanreotide or vapreotide,^51^ where it enhanced SST_5_ binding, Thr^6^ was replaced by Val^6^ either without modifying the C-terminus (Phe(4NO_2_)^1^/dCys^2^, Val^6^, 5) or in combination with an aromatic C-terminal residue (Phe(4NO_2_)^1^/dCys^2^, Val^6^, dTyr^8^-NH_2_, 13). Contrary to expectations, this substitution resulted in loss of SST_5_ affinity and reduced SST_2_ affinity compared to the corresponding Thr^6^-containing analogs (4 and 12, respectively), while leaving ligand functionality unchanged.

Overall, these results indicate that, within the DOTA-NOC scaffold, simple inversion of chirality at positions 1 and 2 does not promote SST_2_ antagonism but instead impairs affinity. Incorporation of an aromatic residue at the C-terminus, like dTyr-NH_2_ in combination with inverted chirality – features commonly associated with potent SST_2_-specific antagonists, like DOTA-JR11 or DOTA-LM3 – is critical for inducing SST_2_ antagonism, at the cost of SST_5_ recognition within this scaffold.

### 2.4 *In vivo* evaluation

To assess how the above modifications translate *in vivo*, the most promising ligands were evaluated by SPECT/CT imaging and biodistribution studies.

#### 2.4.1 SPECT/CT imaging

SPECT/CT imaging of the six selected radioligands was performed in HEK-SST_2_ tumor-bearing mice, 1 hour after administration of the radioligand (200 pmol/100 μL/10–15 MBq) and compared to [^177^Lu]Lu-DOTA-NOC (Fig. 4). All six radioligands were able to delineate and visualize the SST_2_-expressing tumors, similarly to [^177^Lu]Lu-DOTA-NOC. By visual assessment, the tumor uptake seemed lower for ^177^Lu-3, and comparable for ^177^Lu-11, ^177^Lu-14 and [^177^Lu]Lu-DOTA-NOC.

**Fig. 4:**
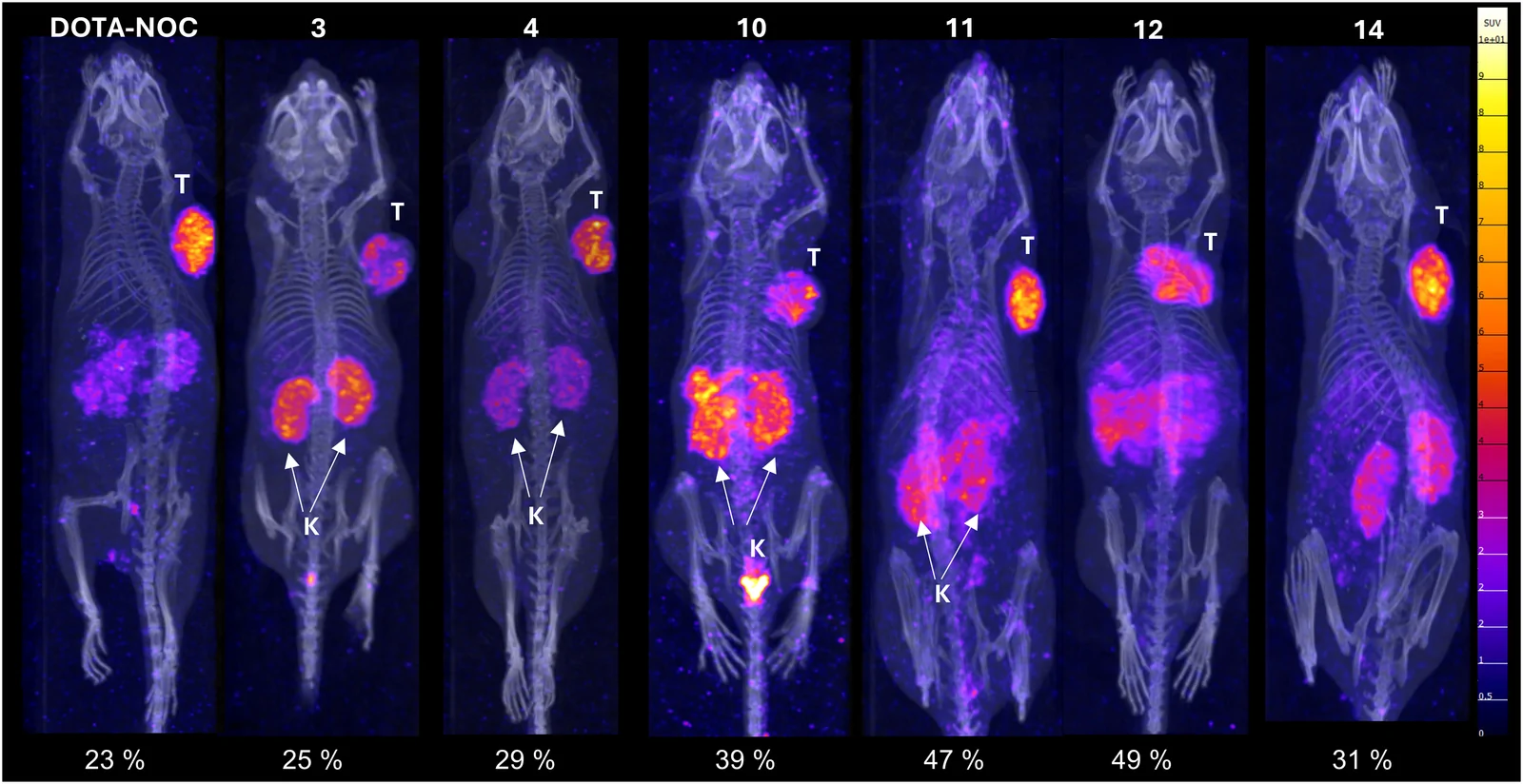
SPECT/CT images of HEK-SST_2_ tumor-bearing mice at 1 h p.i. of the ^177^Lu-labeled ligands (200 pmol, 10–15 MBq). The maximum intensity projection (MIP) images are adjusted in the same scale (SUV 10) for direct comparison. The remaining activity in the body after 1 h is mentioned as % of the injected activity at the bottom for each radioligand, T: tumor, K: kidneys.

SPECT/CT images of the four selected radioligands that demonstrate measurable affinity for SST_5_ were acquired in HEK-SST_5_ tumor-bearing mice, 1 hour post injection and compared to [^177^Lu]Lu-DOTA-NOC (Fig. 5). In HEK-SST_5_ xenografts, ^177^Lu-10 was the only radioligand with a clear tumor visualization, and an uptake much higher than that of [^177^Lu]Lu-DOTA-NOC. Of note, cell line-specific factors may limit direct quantitative comparison between the *in vitro* CHO-SST_5_ and *in vivo* HEK-SST_5_ data. Nevertheless, ligand 10 showed both the highest SST_5_ affinity and the most pronounced uptake in HEK-SST_5_ xenografts, supporting consistency between the observed *in vitro* and *in vivo* trends.

**Fig. 5:**
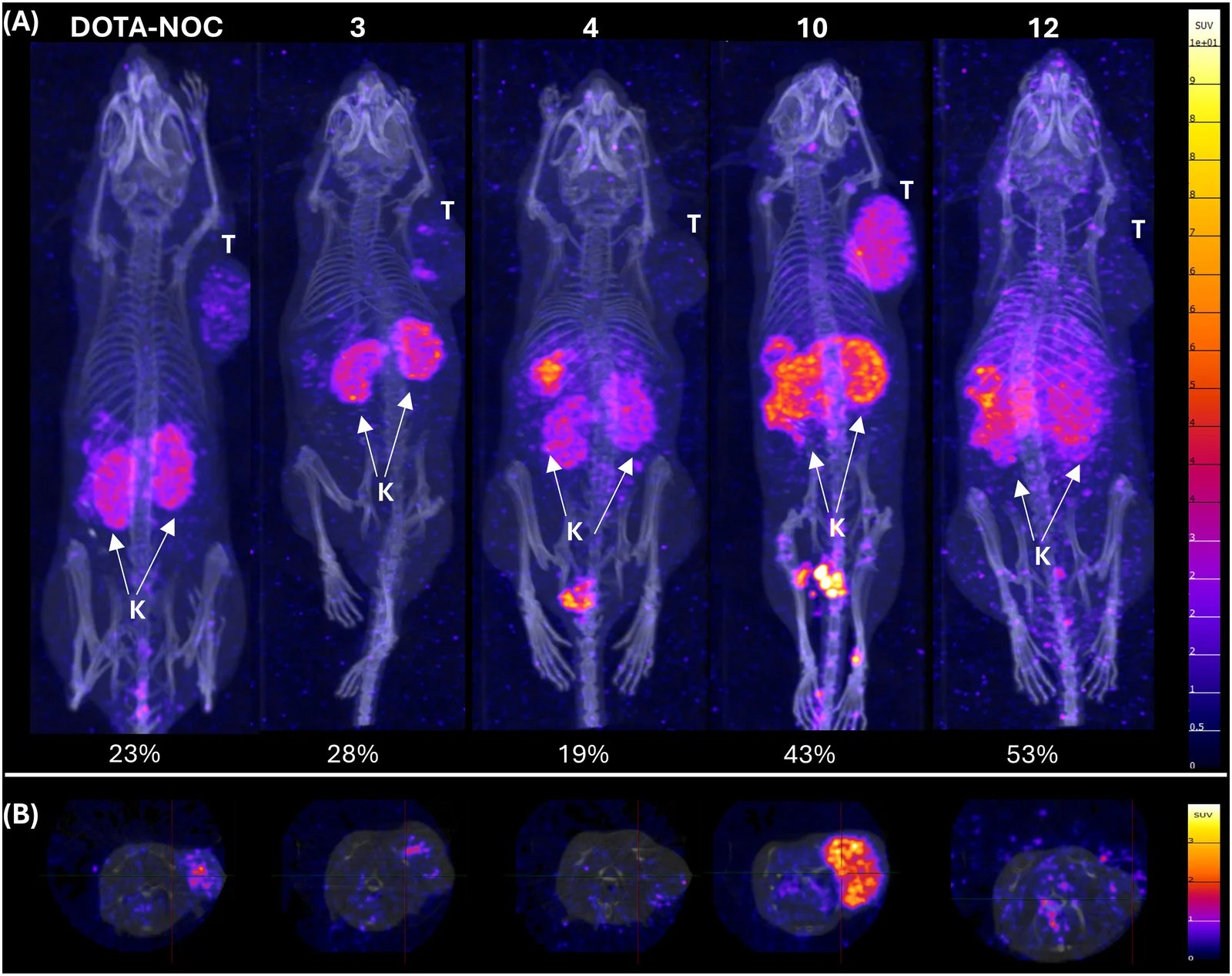
SPECT/CT images of HEK-SST_5_ tumor-bearing mice at 1 h p.i. of ^177^Lu-DOTA-ligands (200 pmol, 10–15 MBq). Images have been adjusted in the same scale (SUV 10 for whole body scan (A), transaxial view of the tumor (SUV 4, B)) for direct comparison. The remaining activity in the body of the mice after 1 h is mentioned below the whole-body images. T: tumor, K: kidneys.

An important observation was the total body distribution of each radioligand. While kidney uptake was observed for all the radioligands, suggesting renal clearance as the primary excretion pathway, ^177^Lu-10, ^177^Lu-11 and ^177^Lu-12 displayed additionally pronounced abdominal uptake and high residual total-body activity. This pattern resembles the biodistribution profile reported for SST_2_-selective antagonists such as [^177^Lu]Lu-DOTA-JR11.^48^ The abdominal uptake of [^177^Lu]Lu-DOTA-JR11 was attributed to the specific uptake of the radioligand in somatostatin receptor expressing tissues, like the stomach and pancreas, as a result of its high SST_2_-affinity. In other words, this uptake may indirectly reflect efficient SST_2_-targeting *in vivo*.

In contrast to all other ligands, ^177^Lu-10 demonstrated robust accumulation and clear visualization in both SST_2_- and SST_5_-expressing tumor models. Importantly, in the SST_5_ model, ^177^Lu-10 was the only radioligand that enabled clear tumor visualization, including [^177^Lu]Lu-DOTA-NOC. Based on the imaging data and considering all relevant properties, ^177^Lu-10 was selected for quantitative biodistribution studies and compared with the reference compound [^177^Lu]Lu-DOTA-NOC.

#### 2.4.2 Quantitative biodistribution of ^177^Lu-10 *vs.* [^177^Lu]Lu-DOTA-NOC

Biodistribution studies at 1 h, 4 h and 24 h p.i. were performed in HEK-SST_2_ and HEK-SST_5_ xenografted mice injected with 10 pmol/100 μL/0.3–0.6 MBq radioligand (Table 2).

**Table 2:** Biodistribution results of [^177^Lu]Lu-DOTA-NOC and ^177^Lu-10 (10 pmol/0.3–0.6 MBq) in HEK-SST_2_ and HEK-SST_5_ xenografted nude mice at 1, 4 and 24 hours post-injection. Non-tumor organ and blood uptake values were pooled from both xenograft groups because whole-body biodistribution was the same between the models. The results are expressed % IA g^−1^ ± SD (*n* = 4–8 mice per group)

Organ	[^177^Lu]Lu-DOTA-NOC			^177^Lu-10		
	1 h	4 h	24 h	1 h	4 h	24 h
Blood	0.60 ± 0.10	0.09 ± 0.04	0.02 ± 0.00	1.31 ± 0.37	0.13 ± 0.04	0.04 ± 0.01
Heart	0.37 ± 0.06	0.15 ± 0.03	0.06 ± 0.01	1.56 ± 0.42	0.29 ± 0.05	0.14 ± 0.02
Lung	7.10 ± 0.96	6.81 ± 1.78	3.11 ± 1.21	13.39 ± 2.39	14.53 ± 3.62	8.36 ± 3.10
Liver	0.80 ± 0.07	0.50 ± 0.06	0.21 ± 0.04	2.11 ± 0.54	1.36 ± 0.27	0.82 ± 0.09
Pancreas	17.62 ± 2.88	9.40 ± 1.17	2.90 ± 0.65	32.04 ± 4.96	14.32 ± 3.10	9.68 ± 1.22
Spleen	0.74 ± 0.11	0.54 ± 0.09	0.37 ± 0.11	1.98 ± 0.47	1.15 ± 0.33	0.75 ± 0.11
Stomach	13.23 ± 2.95	10.77 ± 1.79	6.28 ± 0.84	24.68 ± 4.22	16.99 ± 4.59	12.82 ± 1.03
Intestine	2.97 ± 1.53	3.13 ± 0.71	1.89 ± 0.59	5.47 ± 1.28	5.40 ± 0.88	4.46 ± 0.79
Adrenal	3.47 ± 0.49	3.86 ± 0.98	2.03 ± 0.65	6.00 ± 2.18	5.18 ± 1.40	5.04 ± 1.70
Kidney	7.91 ± 1.05	6.35 ± 1.05	2.55 ± 0.56	17.67 ± 4.46	13.10 ± 2.34	7.69 ± 0.78
Muscle	0.13 ± 0.04	0.05 ± 0.02	0.02 ± 0.02	0.39 ± 0.12	0.07 ± 0.01	0.04 ± 0.01
Femur	0.95 ± 0.27	0.78 ± 0.13	0.55 ± 0.23	2.49 ± 0.47	1.78 ± 0.54	1.01 ± 0.15
SST_2_-tumor	20.17 ± 1.16	20.33 ± 2.57	11.64 ± 1.34	19.61 ± 3.23	16.14 ± 4.25	6.20 ± 1.57
SST_5_-tumor	2.18 ± 0.29	0.78 ± 0.20	0.59 ± 0.13	10.49 ± 0.47	3.07 ± 0.50	1.42 ± 0.16

The overall quantitative biodistribution of ^177^Lu-10 was comparable to that of other radiolabeled somatostatin analogs, showing accumulation in SST_2_ expressing tissues such as the stomach, pancreas, and kidneys. In the head-to-head comparison, the uptake in SST_2_-expressing tumors was comparable between ^177^Lu-10 and [^177^Lu]Lu-DOTA-NOC at 1 h p.i. (19.61 ± 3.23 *vs.* 20.17 ± 1.16 % IA g^−1^, *p* = 0.7585) and at 4 h p.i. (16.14 ± 4.25 *vs.* 20.33 ± 2.57 % IA g^−1^, *p* = 0.1192). However, at 24 h p.i. ^177^Lu-10 showed lower uptake than [^177^Lu]Lu-DOTA-NOC (6.20 ± 1.57 *vs.* 11.64 ± 1.34 IA g^−1^, *p* = 0.0043). On the other hand, ^177^Lu-10 revealed a markedly higher uptake in SST_5_-expressing tumors compared to [^177^Lu]Lu-DOTA-NOC, with almost 5-fold higher uptake at 1 h p.i. (10.49 ± 0.47 *vs.* 2.18 ± 0.29 % IA g^−1^, *p* < 0.0001), approximately 4-fold higher uptake at 4 h p.i. (3.07 ± 0.50 *vs.* 0.78 ± 0.20 % IA g^−1^, *p* < 0.0001) and 2.4-fold higher uptake at 24 h p.i. (1.42 ± 0.16 *vs.* 0.59 ± 0.13 % IA g^−1^, *p* < 0.001). Significantly higher accumulation was also observed for ^177^Lu-10 in the somatostatin receptor expressing tissues at 1 h p.i., including the stomach (∼1.9-fold, *p* = 0.0001), the pancreas (∼1.8-fold, *p* = 0.0001), and the kidneys (∼2.2-fold, *p* = 0.0001). The longer circulation in the blood and the higher accumulation in the liver might be explained by the higher lipophilicity of ^177^Lu-10*vs.* [^177^Lu]Lu-DOTA-NOC (log *D* = −1.02 ± 0.17 *vs.* −1.61 ± 0.19, respectively).

The specificity of ^177^Lu-10 was confirmed by biodistribution in HEK xenograft-bearing mice performed at 1 h p.i. (Table S5). As expected, the overall biodistribution profile remained unchanged, except for tumor uptake, which was <1% IA g^−1^ of the injected activity. Tumor uptake was reduced by 96% and 92% compared with the accumulation observed in HEK-SST_2_ and HEK-SST_5_ tumors, respectively.

In comparison to SST_2_-specific radioligands, ^177^Lu-10 showed high early SST_2_-tumor uptake at 1 and 4 h p.i., declining to approximately 40% by 24 h. This profile resembles that of SST_2_ agonists such as [^177^Lu]Lu-DOTA-TATE,^48^ and is less favorable than that typically reported for SST_2_ antagonists like [^177^Lu]Lu-DOTA-JR11^48^ and [^177^Lu]Lu-DOTA-LM3.^54^ On a whole-body basis, ^177^Lu-10 showed higher retention in several normal organs, particularly kidneys and physiologically SST-expressing tissues, such as the pancreas, stomach, and adrenals up to 24 h, than [^177^Lu]Lu-DOTA-TATE. This pattern resembles that of SST_2_-specific antagonists [^177^Lu]Lu-DOTA-JR11^48^ and [^177^Lu]Lu-DOTA-LM3,^54^ which generally exhibit more persistent whole-body activity than agonists. Notably, the peptide mass used for biodistribution (10 pmol) was substantially lower than that used for imaging (200 pmol), potentially affecting uptake in both tumors and SST-positive normal tissues. A similar “mass effect” has been described for the high-affinity SST_2_ antagonist [^177^Lu]Lu-DOTA-JR11, for which increasing the administered mass from 10 to 200 pmol led to more rapid receptor saturation in normal organs than in tumours.^48^ This effect may be particularly relevant for lower-expressing targets or lower-affinity ligands and could therefore have influenced SSTR_5_ targeting in the imaging experiments.

As discussed earlier, the higher lipophilicity of ^177^Lu-10*vs.* [^177^Lu]Lu-DOTA-NOC might increase the whole-body radiation dose, as has been reported for [^177^Lu]Lu-DOTA-NOC *vs.* the more hydrophilic [^177^Lu]Lu-DOTA-TATE.^55^ On the other hand, the dual SST_2_/SST_5_ targeting of DOTA-NOC allowed [^68^Ga]Ga-DOTA-NOC to detect lesions that express SST_5_-dominant or mixed-subtype patterns, which are sub-optimally visualized with [^68^Ga]Ga-DOTA-TATE alone.^56^ [^68^Ga]Ga-DOTA-NOC is recognized in guidelines as a first-line PET radioligand for neuroendocrine neoplasms, interchangeable with [^68^Ga]Ga-DOTA-TOC and [^68^Ga]Ga-DOTA-TATE, and has already been used to image many patients in routine clinical practice.^57,58^ Its high affinity for both SST_2_ and SST_5_ may therefore provide added value when broader receptor coverage or detection of SST_5_-rich tumors is required. In this context, the markedly higher uptake of ^177^Lu-10 in SST_5_-expressing tumors compared with [^177^Lu]Lu-DOTA-NOC is of key relevance. Nevertheless, the elevated retention in normal organs may reduce the therapeutic window by increasing normal-tissue absorbed doses and the risk of organ-specific toxicity, particularly in dose-limiting tissues. Future optimization could include modifying the ligand's pharmacokinetic properties or exploiting the “mass effect”,^48^ discussed above.

## 3. Conclusion

This study provides important insights into the structural determinants of octapeptide-based ligands governing SST_2_ and SST_5_ affinity, selectivity, and antagonism. The inversion of chirality at positions 1 and 2 (l^1^/d^2^), together with the presence of an aromatic C-terminal residue, appears to be essential for achieving SST_2_ antagonism within the DOTA-NOC scaffold, as exemplified by 11. In contrast, the same chirality inversion is poorly tolerated for SST_5_ affinity, which could only be partially restored by introducing substituted aromatic residues such as Phe(4NO_2_) at position 1. Furthermore, SST_5_ antagonism was observed only when Cys at position 2 was maintained in the l-configuration. These findings indicate that the structural requirements for SST_2_ antagonism are largely incompatible with those governing SST_5_ antagonism in the DOTA-NOC scaffold. Among the ligands investigated, 10, combining the d^1^/l^2^ configuration with dTyr^8^-NH_2_, displayed the most favorable profile, exhibiting low nanomolar affinity for both SST_2_ and SST_5_ together with selective SST_5_ antagonism. This profile positions 10 as a promising alternative to SST_2_-selective ligands, with the potential to improve visualization and targeting of tumors in which SST_5_ expression plays a complementary or predominant role.

## 4. Experimental section

### 4.1 Materials and methods

The Nα-fluorenylmethoxycarbonyl (Fmoc)-protected amino acids, Rink Amide resin (0.67 mmol g^−1^) and preloaded *O-t*-butylthreoninol 2-chlorotrityl resin (0.5 mmol g^−1^) were purchased from (Iris Biotech GmbH, Marktredwitz, Germany) or Merck Novabiochem (Darmstadt, Germany), respectively. DOTA-tris(*t*Bu)ester (DOTA(*t*Bu)_3_) was obtained from CheMatech (Dijon, France). Fmoc-Phe(4Cl)-OH, ethyl cyanohydroxyiminoacetate (Oxyma) and *N*,*N*′ diisopropylcarbodiimide (DIC) were obtained from Bachem (Bubendorf, Switzerland) and CEM (Matthews, USA). [^177^Lu]LuCl_3_ (non-carrier added) was provided by ITM (Munich, Germany). All other reagents and solvents were purchased from Acros Organics (Geel, Belgium), Merck Millipore (Darmstadt Germany) and Bioconcept (Allschwil, Switzerland), and used without further purification. Detailed instrumentation is reported in SI.

### 4.2 Peptide synthesis and DOTA-conjugation

All the peptides were synthetized following the Fmoc/*t*Bu strategy, either manually or assembled on the automated microwave peptide synthesizer Liberty Blue (CEM, Charlotte, NC, USA). Rink amide or 2-chlorotrityl resins were used according to the peptide structure. The coupling of the amino acids (four equivalents) was performed using DIC (*N*,*N′*-diisopropylcarbodiimide, 4 eq.) as an activator and oxyma (4 eq.) as a base, while Fmoc deprotection occurred in the presence of 20% piperidine in DMF (dimethylformamide). The formation of the disulfide bond (Cys–Cys cyclization) took place on resin, by incubation with 5 eq. of iodine diluted in DMF for 2 h. Following the addition of the last amino acid, Fmoc was deprotected and DOTA(*t*Bu)_3_ (2 eq.) with HATU (2-(7-azabenzotriazol-1-yl)-*N*,*N*,*N*′,*N*′-tetramethyluronium hexafluorophosphate, 2 eq.) and DIPEA (*N*,*N*′ diisopropylethylamine, 4 eq.) was dissolved in 3 mL DMF and added to the resin. The peptides were cleaved in 95% (v/v) TFA (trifluoroacetic acid), 2.5% (v/v) TIS (triisopropylsilane), and 2.5% (v/v) H_2_O overnight. The peptide residue was precipitated and washed with cold diethyl ether by centrifugation. The crude peptides were purified using the semi-preparative LC-MS-2020 Shimadzu system mentioned above and characterized by LC-MS.

### 4.3 Radiolabeling

The ^177^Lu-labeled ligands were obtained by incubating 2 nmol of the ligand in 200 μL of ammonium acetate buffer (0.4 M, pH 5.0) with [^177^Lu]LuCl_3_ (20–60 MBq nmol^−1^, depending on the planned experiment) for 30 min at 95 °C. A small aliquot was withdrawn from the radiolabeled solution, added to Ca-DTPA (calcium diethylenetriamine pentaacetate, 0.1 M, 50 μL) and injected on the radio-RP-HPLC for quality control. To obtain a structurally homogenous ligand for the saturation binding assay, an equivalent amount of ^nat^LuCl_3_ × 5H_2_O (3 × 10^−4^ M) was added and the radiolabeling solution was further incubated for 30 min at 95 °C. The reaction solution was then diluted with saline to obtain the final dilutions.

### 4.4 log *D* determination

Lipophilicity was determined using the “shake-flask” method. 10 μL of the radioligand (10 pmol) were added to a pre-saturated mixture of 500 μL of octanol and 500 μL of phosphate-buffered saline (PBS), pH = 7.4, in triplicate. The solutions were then vortexed for 3–5 min, shaken for 1 h and centrifuged for 10 min at 10 000 rpm. Three aliquots of 100 μL were withdrawn from each phase and measured in the γ-counter. The distribution coefficient (log *D*_pH=7.4_) was calculated as the average log ratio value of the radioactivity in the organic and PBS fractions (log *D*_pH=7.4_ = log_10_ (counts in octanol phase/counts in aqueous phase)).

### 4.5 *In vitro* evaluation

#### 4.5.1 Cell culture

The human embryonic kidney HEK-293 cell line expressing the T7-epitope tagged human SST_2_ receptor (HEK-SST_2_) and HEK-SST_5_ were cultured at 37 °C and 5% CO_2_ in Dulbecco's modified Eagle medium (DMEM) containing 10% fetal bovine serum (FBS), 100 U mL^−1^ penicillin, 100 mg mL^−1^ streptomycin, 200 μmol mL^−1^l-glutamine, and 500 mg mL^−1^ G418. The level of somatostatin receptor expression was between 800 and 1200 fmol mg^−1^ membrane protein.^59^ For the *in vitro* cell experiments, the plates were pre-treated with a solution of 10% (v/v) poly-l-lysine to promote cell attachment. HEK-SST_2_ cells were then seeded at a density of 1 × 10^6^ cells per well in 6-well plates and incubated overnight in DMEM with 1% FBS to obtain good cell adherence. Cell membrane preparation is described in SI.

#### 4.5.2 Cellular uptake on intact cells

On the day of the experiment, the cells were incubated with the radioligand (2.5 nM) for 1, 2 and 4 hours at 37 °C. The non-specific cellular uptake was determined by using non-transfected HEK cells in selected wells. The internalization was stopped by removing the medium and washing the cells twice with ice-cold PBS. This represented the free fraction. The cell surface-bound (acid releasable) fraction was collected with an acidic glycine solution (pH = 2.8) while the internalized fraction was obtained collecting the cells with a basic solution of NaOH (1 M). The internalized and cell surface bound fractions were quantified using a γ-counter and represented as a percentage of the total activity added per well.

#### 4.5.3 Inhibition assay (IC_50_)

The half maximal inhibitory concentration (IC_50_) values of all non-metallated ligands were determined by competition binding with ^125^I-Tyr-SS-14 (Chelatec, France) on HEK-SST_2_ and CHO-SST_5_ membranes (Perkin Elmer, CHO-K1, human Somatostatin SST_5_ receptor, product no.: ES-522-M400UA). The procedure is detailed in SI.

#### 4.5.4 Saturation binding assay

Saturation binding assays were conducted in HEK-SST_2_ and CHO-SST_5_ cell membranes as described in detail in the SI.

#### 4.5.5 Functional phosphorylation assay (7TM phosphorylation assay)

Functional phosphorylation assay (7TM Phosphorylation Assay) of HA-tagged SST_2_ or SST_5_ was assessed as described in Kaufmann *et al.* (2022).^60^ A brief description of the procedure is reported in the SI.

### 4.6 *In vivo* evaluation

#### 4.6.1 Animal models

Animal experiments were conducted in accordance with the Swiss animal welfare laws and regulations under license number 2799 granted by the Veterinary Office (Department of Health) of the Cantonal Basel-Stadt. Female athymic nude-Foxn1^nu^/Foxn1^+^ mice (Envigo, The Netherland), 4–6 weeks old, were inoculated subcutaneously either with 10^7^ HEK-SST_2_ cells (SST_2_-positive xenografts) or HEK-SST_5_ (SST_5_-positive xenografts), freshly suspended in 100 μL sterile PBS, in the shoulder. The xenografted mice were monitored, and the tumors were allowed to grow for 3–4 weeks, reaching a volume of 100–200 mm^3^.

#### 4.6.2 SPECT/CT imaging

HEK-SST_2_ or HEK-SST_5_ xenografted mice were injected intravenously with the radioligand (200 pmol/100 μL/10–15 MBq) *via* the tail vein. The mice were euthanized at 1 h post-injection (p.i.) and imaged supine, head first, using a SPECT/CT system dedicated to small animals (NanoSPECT/CT™ Bioscan Inc.). A helical CT scan was acquired with the following parameters: current, 177 mA; voltage, 45 kVp; pitch, 1. A helical SPECT scan was acquired using multipurpose pinhole collimators (APT1), 20% energy window width centered symmetrically over the 208 and 113 keV gpeaks of ^177^Lu, 24 projections, and 1200 s per projection. CT and SPECT images were reconstructed and filtered using the manufacturer's algorithm, resulting in a pixel size of 0.3 mm for the SPECT and of 0.2 mm for the CT.

#### 4.6.3 Biodistribution studies

Quantitative biodistribution studies were performed in HEK-SST_2_ and HEK-SST_5_ xenografts for selected ligands. The radioligands were injected intravenously *via* the teil veil (10 pmol/100 μL/0.4–0.5 MBq) and at pre-selected time points, the mice were euthanized by CO_2_ asphyxiation. Blood and organs of interest were collected, blotted dry, weighed and counted in a γ-counter. The samples were counted against a suitably diluted aliquot of the injected solution as the standard. The results are expressed as percentage of injected activity per gram (% IA/g) and represent the mean ± standard deviation (SD). HEK-293 xenografts (SST-negative) were used to assess the specificity of the lead ligand, ^177^Lu-10, one hour after injection of 10 pmol/0.4 MBq.

### 4.7 Ethical statement

All animal procedures were performed in accordance with the Guidelines for Care and Use of Laboratory Animals of University of Basel and approved by the Veterinary Office (Department of Health) of the Cantonal Basel-Stadt, Switzerland.

### 4.8 Statistical analysis

The statistical analysis was performed using the program GraphPad Prism (version 10). The *p*-values were calculated *via* GraphPad using a *t* test unpaired analysis, with *p* < 0.05 considered statistically significant.

## Author contributions

Fabienne Spinnler: methodology, validation, investigation, formal analysis, visualization, writing – original draft. Luigi Del Pozzo: investigation, validation. Lennart Bohrmann: investigation, validation. Sandra Zanger: investigation, validation. Nina Kathleen Blum: validation, formal analysis, visualization. Monique Brendel: investigation, validation. Stefan Schulz: methodology, supervision, project administration. Rosalba Mansi: conceptualization, methodology, formal analysis, visualization, supervision, project administration, writing – original draft, writing –review and editing. Melpomeni Fani: conceptualization, resources, supervision, project administration, funding acquisition, writing – review and editing. All authors read and approved the final manuscript.

## Conflicts of interest

Melpomeni Fani and Stefan Schulz received research funding from ITM (Munich, Germany) related to this work. Stefan Schulz is the founder and scientific advisor of 7TM Antibodies GmbH, Jena, Germany. All other authors declare that they have no known competing financial interests or personal relationships that could have appeared to influence the work reported in this paper.

## Supplementary Material

MD-017-D6MD00646A-s001

## Data Availability

The data supporting this article are available in the manuscript and its supplementary information (SI). Supplementary information is available. See DOI: https://doi.org/10.1039/d6md00646a.
