# Supplementary material for: Development of dual SST2/SST5 targeting radioligands through rational structural design
Source: RSC Med Chem. 2026 Oct 5;17(10):5101–12. doi: 10.1039/d6md00646a (PMC13637744; doi:10.1039/d6md00646a)
Supplement: Supplementary file 1 [file MD-017-D6MD00646A-s001.pdf]

Supplementary Information (SI) for RSC Medicinal Chemistry.
This journal is © The Royal Society of Chemistry

Supplementary Information

Development of dual SST_2/SST_5 targeting radioligands through rational structural design

Fabienne Spinnler,[a] Luigi Del Pozzo,[a] Lennart Bohrmann,[a] Sandra Zanger,[a] Nina Kathleen Blum,[b] Monique Brendel,[b] Stefan Schulz,[b] Rosalba Mansi*[a] and Melpomeni Fani*[a]

[a] Division of Radiopharmaceutical Chemistry, Department Theragnostics, University Hospital Basel, University of Basel, Basel 4031, Switzerland

[b] Institute of Pharmacology and Toxicology, University Hospital Jena, Friedrich Schiller University, Jena 07747, Germany

*Corresponding authors:

Prof. Melpomeni Fani and Dr. Rosalba Mansi

University Hospital Basel, Switzerland (CH)

Phone +41(0)61 556 58 91

E-Mail: melpomeni.fani@usb.ch, rosalba.mansi@usb.ch

Experimental procedures

Analytical methods and instrumentation. Quantitative γ-counting was carried out on a COBRA 5003 γ-system well counter from Packard Instruments (Meriden, USA). Analytical liquid chromatography mass spectrometry (LC-MS) was performed using an LC-MS-2020 Shimadzu instrument equipped with a Phenomenex Jupiter Proteo 90 Å, C12 column (250 x 4.6 mm, 4 μm particle size). The gradient was set to 25-95% eluent B for 15 minutes at a wavelength of 214 nm, with eluent A = H_2O (+ 0.1% trifluoroacetic acid (TFA)) and eluent B = Acetonitrile (ACN) (+ 0.1% TFA) flow rate: 1 mL/min. For purification purposes, the same HPLC instrument as above mentioned was used with a semi-preparative Phenomenex Jupiter Proteo 90 Å, C12 column (150 x 10 mm, 5 μm particle size), and a gradient 15-65% eluent B was used during 15 min at a flow rate of 5 mL/min. For the quality control of the radioligands, high-performance liquid chromatography (HPLC) was performed on the HPLC-Agilent system equipped with a Berthold LB509 γ-detector using a Phenomenex Jupiter Proteo, C12 column (250 x 4.6 mm, 4 μm particle size), gradient B = 15-65% eluent B in 15 min, flow rate: 2 mL/min.

Cell membrane preparation. The HEK-SST_2 cells were grown to confluence, mechanically disaggregated, washed with PBS buffer (pH 7.0), and re-suspended in homogenization buffer (tris (hydroxymethyl) aminomethane (Tris) 20 mM, Ethylenediaminetetraacetic acid (EDTA) 1 mM, sucrose 0.25 M, bacitracin 1 mg/mL, soybean trypsin inhibitor 0.1 mg/mL, and phenylmethylsulfonyl fluoride (PMSF) 0.125 mg/mL, pH 7.5). Cells were sonicated on ice using Ultra-Turrax to lyse the cells and the cells suspension was centrifuged at 1'000 rpm for 10 min at 4°C. The supernatant was collected in centrifuge tubes (Beckman Coulter Inc., Brea, USA) and this procedure was repeated four times. The collected supernatant (membrane proteins) was centrifuged in an ultra-centrifuge (Beckman Coulter Inc., Brea, USA) at 4°C for 55 min at 20'000 rpm. Afterwards, the pellet was re-suspended in ice-cold 4-(2-hydroxyethyl)-1-piperazineethanesulfonic acid (HEPES) buffer (10 mM, pH 7.5), aliquots were prepared (2000 mg/mL), and stored at -80°C. The protein concentration of the membrane solution was determined using the Bradford method with bovine serum albumin as the standard.

CHO-SST_5 membranes were purchased by Perkin Elmer (WA, USA).

Inhibition assay (IC_{50}). HEK-SST_2 or CHO-SST_5 cell membrane suspensions, corresponding to 10 μg/well, were incubated for 1 h at 37°C in 96-wells plates with increasing concentrations of each ligand in the presence of ^{125}I-Tyr-SS-14 (0.5 nM). Final ligand concentrations ranged from 0.01 to 100 nM for SST_2 and from 0.01 to 1000 nM for SST_5). For the non-specific series, SS-14 (1 μM) was added in three selected wells. Rapid

filtration followed on a Brandel 48-well Cell Harvester using GF/C filter (Whatman), pre-soaked with 0.1% PEI solution. The filter was washed with 3 x 1 mL 20 mM HEPES. Filter activity was measured on an automatic gamma-counter. Non-specific binding is defined as the amount of activity binding in the presence of 1000-fold excess of SS-14. The data were analysed by GraphPad Prism 10 Software and the IC_{50} values were determined using the 'log(inhibitor) vs response' equation.

Saturation binding assay. The membrane suspensions (10 μg/well) were incubated for 1h at 37°C in binding buffer with increasing concentrations of each radioligand (final concentration range: 0.075-10 nM; 30 μL per well). To determine non-specific binding, selected wells received a 1000-fold excess of blocking agent (SS-14 or DOTA-/NODAGA-JR11). Following incubation, samples were harvested on a Brandel 48-well Cell Harvester using GF/C filter (Whatman), pre-soaked with 0.1% polyethyleneimine which helps the membranes to stick to the filter. Filters were washed with HEPES buffer (50 mM) to remove the fraction of the radioligand not bound to the membrane. Finally, the filters were collected in different tubes and the activity measured using a γ-counter. The data were analyzed using GraphPad Prism 10 Software. Because some radioligands showed substantial binding, ligand depletion was considered in the saturation-binding analysis. Free radioligand activity was estimated by subtracting bound activity from the total activity added. Specific binding (total binding minus nonspecific binding) was then plotted against this estimated free activity. Because free radioligand concentrations were not measured directly, these values represent estimates rather than a formal correction for ligand depletion.

Functional phosphorylation assay (7TM Phosphorylation Assay). HEK293 cells stably expressing the receptors were seeded in poly-L-lysine-coated 96-well plates and treated at ~95% confluency with increasing concentrations of the test compounds. For antagonist assays, cells were preincubated with the compound (30 min, 37°C) before stimulation with 1 μM SS-14 for 30 min. Cells were lysed using a detergent buffer supplemented with protease and phosphatase inhibitors. Lysates were split into duplicate wells for parallel detection of phosphorylated and total receptor levels. Receptors were immunoprecipitated with anti-HA magnetic beads and washed with PBS-T. Phosphorylation was detected using phosphosite-specific antibodies: pS341/pS343-SST2 or pT333-SST5 (7TM Antibodies). Total receptor levels were measured with a rabbit anti-HA antibody (7TM Antibodies). Following overnight incubation, an HRP-linked secondary antibody (Cell Signaling Technology) and Super Aqua Blue substrate (ThermoFisher) were used to induce color reaction. This reaction was stopped with 0.625 M oxalic acid and absorbance was measured at 405 nm (FlexStation3). Data were acquired using SoftMax Pro 5.4, normalized to internal controls.

RP-HPLC of all tested ligands

Table S1. Analytical data for all tested ligands. For each ligand, the retention time (t_R), purity (%), calculated monoisotopic mass (M_{mi}) and observed ESI-MS mass (m/z) corresponding to the $[M+2H^+]^{2+}$ ion are reported.

	Code	t_r[a] (min)	Purity (%)	M_{mi}[b] (u)	$[M+2H^+]^{2+}$ (u)
Series 1	DOTA-NOC	9.22	97.9	1454.64	728.65
	1	9.03	97.3	1454.64	728.70
	2	9.57	96.8	1454.64	728.70
	3	9.80	97.5	1454.64	728.70
	4	9.18	97.6	1499.62	751.20
	5	10.62	99.3	1497.64	750.30
Series 2	6	8.97	96.4	1467.63	735.30
	7	9.23	97.1	1468.62	735.70
	8	9.75	98.2	1502.58	753.00
	9	10.48	96.9	1511.62	757.25
	10	9.48	95.5	1529.65	766.30
Series 3	11	9.65	98.9	1529.65	766.35
	12	9.70	97.7	1574.63	788.80
	13	10.85	97.9	1572.65	787.80
	14	11.68	96.6	1593.58	798.40
	15	11.93	99.0	1582.56	793.10
	16	11.88	98.1	1597.63	800.60

[a]t_R: Retention time determined by analytical RP-HPLC under conditions described in the Experimental Section, Materials and methods.

[b]M_{mi}: monoisotopic mass

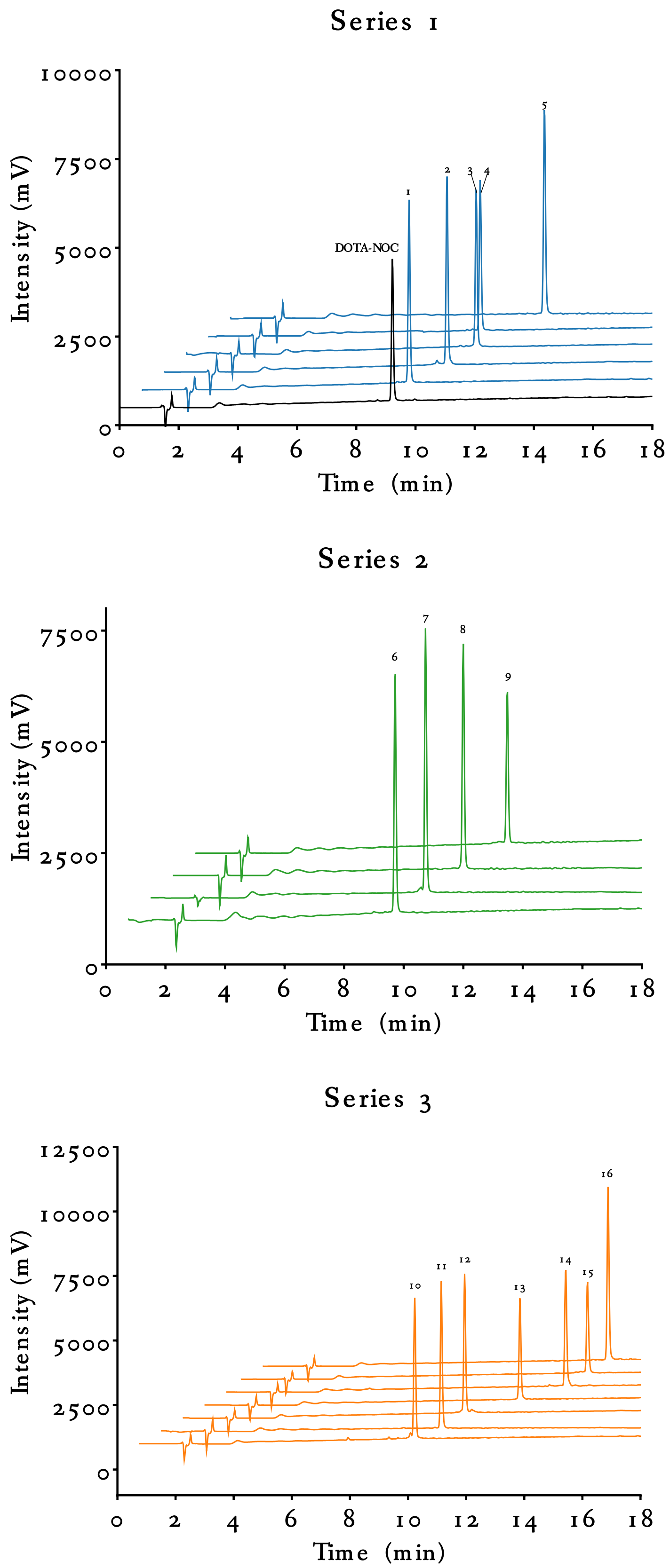

Fig. S1. Representative chromatograms of RP-HPLC analysis for all studied ligands grouped by series.

Radio-RP-HPLC of all tested radioligands

Table S2. Retention time, radiochemical purity and log $D_{O/PBS\,pH\,7.4}$ (the distribution coefficient) of the ^{177}Lu-labeled ligands. The values are reported as average ± standard deviation.

	Code	t_r[a] (min)	Radiochemical purity[b] (%)	log D[c]
Series 1	[^{177}Lu]Lu-DOTA-NOC	9.37	96.6±2.0	-1.61 ± 0.19
	^{177}Lu-**1**	9.82	96.6±2.9	-1.28 ± 0.14
	^{177}Lu-**2**	9.72	96.8±0.8	-1.63 ± 0.06
	^{177}Lu-**3**	11.05	96.3±1.8	-1.89 ± 0.07
	^{177}Lu-**4**	9.83	94.7±2.0	-1.28 ± 0.06
	^{177}Lu-**5**	11.87	93.3±1.6	-0.55 ± 0.04
Series 2	^{177}Lu-**6**	10.18	96.8±1.1	-1.53 ± 0.10
	^{177}Lu-**7**	10.33	95.5±2.8	-1.82 ± 0.08
	^{177}Lu-**8**	10.87	95.0±0.9	-1.26 ± 0.09
	^{177}Lu-**9**	11.55	97.0±1.1	-1.04 ± 0.09
Series 3	^{177}Lu-**10**	9.93	97.2±0.5	-1.02 ± 0.17
	^{177}Lu-**11**	10.52	96.3±0.3	-0.53 ± 0.07
	^{177}Lu-**12**	10.47	94.9±1.2	-0.54 ± 0.05
	^{177}Lu-**13**	12.48	94.5±1.2	0.23 ± 0.08
	^{177}Lu]-**14**	12.42	95.6±0.3	-0.25 ± 0.11
	^{177}Lu]-**15**	12.82	96.5±0.8	0.45 ± 0.05
	^{177}Lu]-**16**	12.72	95.1±2.6	0.94 ± 0.13

[a]t_R: Retention time determined by radio RP-HPLC under the conditions described in the Experimental Section (Materials and methods).

[b]n=3-7; for DOTA-NOC, n = 20 (used as the reference ligand).

[c]n=2 (each in triplicate)

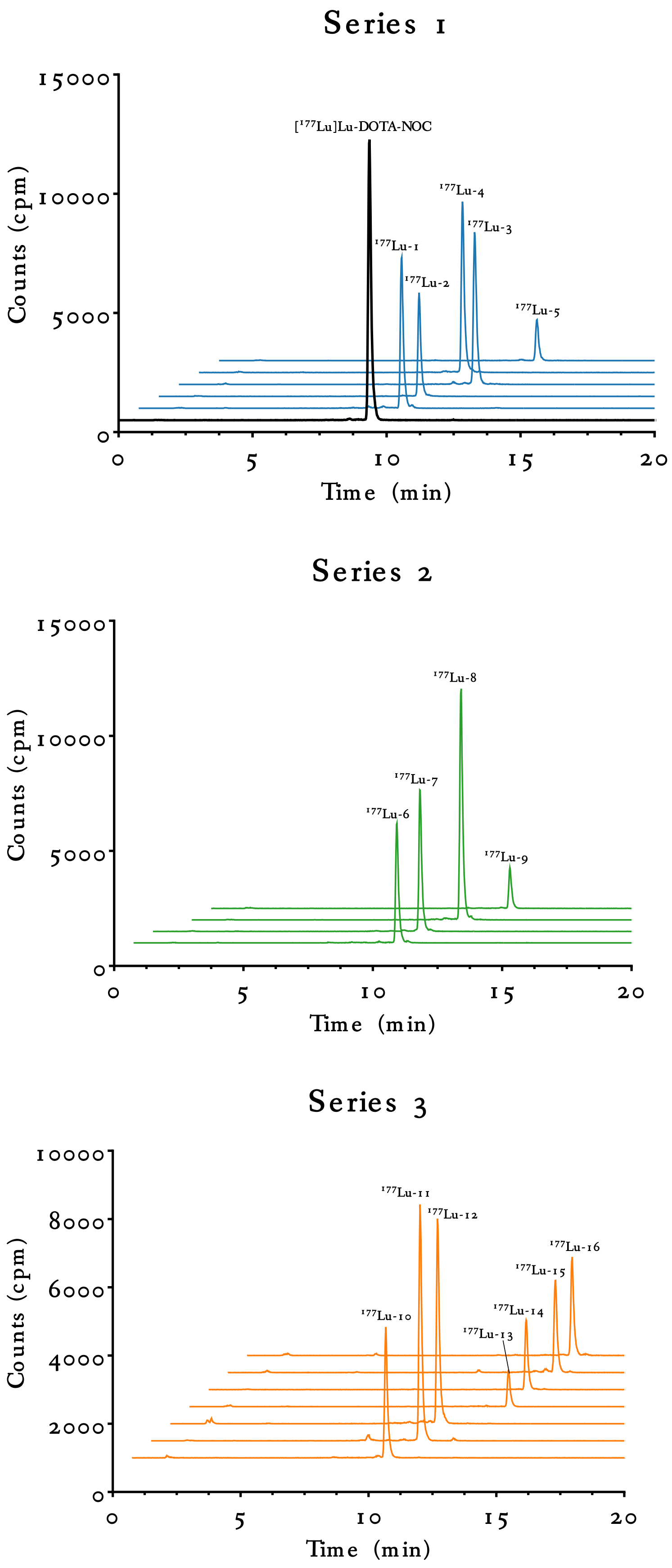

Fig. S2. Representative radiochromatograms of radio-RP-HPLC analysis for all studied ligands grouped by series.

Cellular distribution

Table S3. Cellular distribution of ^{177}Lu-labelled ligands between cell surface (membrane) and internalized fractions in Human Embryonic Kidney (HEK)-SST_2 cells after 1, 2 and 4 hours at 37°C. Each experiment was performed in triplicate. The results are expressed as % (mean ± SD) of applied activity and normalized per million cells.

Code	% of applied activity in SST_2-expressing cells					
	@ 1h		@ 2h		@ 4h	
	Surface bound fraction	Internalized fraction	Surface bound fraction	Internalized fraction	Surface bound fraction	Internalized fraction
[^{177}Lu]Lu-DOTA-NOC	2.5 ± 0.5	34.8 ± 16.9	2.2 ± 0.2	44.4 ± 13.1	1.8 ± 0.6	49.8 ± 9.1
^{177}Lu-**1**	9.9 ± 2.0	2.3 ± 0.5	9.5 ± 1.3	3.4 ± 0.6	7.8 ± 0.4	5.8 ± 1.0
^{177}Lu-**2**	0.1 ± 0.03	0.2 ± 0.03	0.1 ± 0.02	0.2 ± 0.1	0.1 ± 0.02	0.3 ± 0.1
^{177}Lu-**3**	2.3 ± 0.2	54.0 ± 2.2	2.6 ± 0.3	63.3 ± 4.0	3.6 ± 0.5	76.9 ± 1.3
^{177}Lu-**4**	30.3 ± 3.1	5.2 ± 0.2	29.9 ± 2.7	7.1 ± 0.2	30.1 ± 0.2	11.1 ± 0.3
^{177}Lu-**5**	8.0 ± 1.7	2.3 ± 0.1	7.3 ± 1.0	3.6 ± 0.7	6.9 ± 0.8	5.9 ± 1.2
^{177}Lu-**6**	4.3 ± 0.3	1.8 ± 0.1	4.3 ± 0.2	2.6 ± 0.1	4.0 ± 0.2	4.2 ± 0.3
^{177}Lu-**7**	1.9 ± 0.4	7.0 ± 1.3	1.3 ± 0.4	10.4 ± 2.0	2.1 ± 0.7	17.8 ± 0.4
^{177}Lu-**8**	9.4 ± 0.7	6.3 ± 0.1	8.2 ± 0.4	7.6 ± 0.3	6.9 ± 1.0	10.6 ± 0.9
^{177}Lu-**9**	1.8 ± 0.1	1.1 ± 0.1	1.7 ± 0.2	1.5 ± 0.2	1.7 ± 0.2	2.7 ± 0.2
^{177}Lu-**10**	2.7 ± 0.2	68.6 ± 7.3	2.6 ± 0.2	77.6 ± 4.6	2.4 ± 0.4	79.4 ± 2.1
^{177}Lu-**11**	53.6 ± 2.6	9.7 ± 1.0	52.3 ± 1.1	14.4 ± 2.1	47.5 ± 1.4	22.0 ± 1.8
^{177}Lu-**12**	48.3 ± 4.1	11.4 ± 1.5	49.4 ± 3.1	13.5 ± 1.2	47.5 ± 4.1	20.1 ± 1.6
^{177}Lu-**13**	37.2 ± 1.5	16.6 ± 0.8	37.6 ± 3.3	20.6 ± 2.2	33.9 ± 2.1	26.8 ± 3.7
^{177}Lu-**14**	18.8 ± 0.6	28.7 ± 3.1	18.9 ±0.6	31.2 ± 4.9	17.9 ± 0.6	34.8 ± 2.6

^{177}Lu-**15**	11.0 ± 1.0	42.5 ± 3.1	11.2 ± 0.6	47.3 ± 1.6	11.0 ± 0.3	51.9 ± 1.9

Saturation binding studies

Table S4. Dissociation constant (K_D) and maximum number of binding sites (B_{max}) of selected [177/nat]Lu-labeled ligands on HEK-SST_2 and CHO-SST_5 cell membranes after incubation at 37°C.

	Code	SST_2 (nM (95% CI))		SST_5 (nM (95% CI))	
		B_{max}	K_D	B_{max}	K_D
	DOTA-NOC	0.07 (0.06-0.07)	0.04 (0.02-0.05)	0.27 (0.25-0.29)	0.85 (0.66-1.09)
Series 1	3	0.11 (0.10-0.11)	0.07 (0.05-0.09)	0.34 (0.31-0.37)	1.03 (0.78-1.38)
	4	0.16 (0.12-0.21)	1.07 (0.42-2.66)	n.r.d.	n.r.d.
Series 3	10	0.14 (0.13-0.14)	0.06 (0.04-0.09)	0.54 (0.47-0.63)	1.15 (0.71-1.86)
	11	0.20 (0.19-0.21)	0.48 (0.39-0.60)	n.d.	n.d.
	12	0.23 (0.20-0.25)	0.22 (0.14-0.34)	n.r.d.	n.r.d.
	14	0.11 (0.10-0.13)	0.92 (0.54-1.54)	n.d.	n.d.

n.r.d. not reliable data

n.d. not determined as the radioligand did not show any affinity for SST_5 (see **Error! Reference source not found.**).

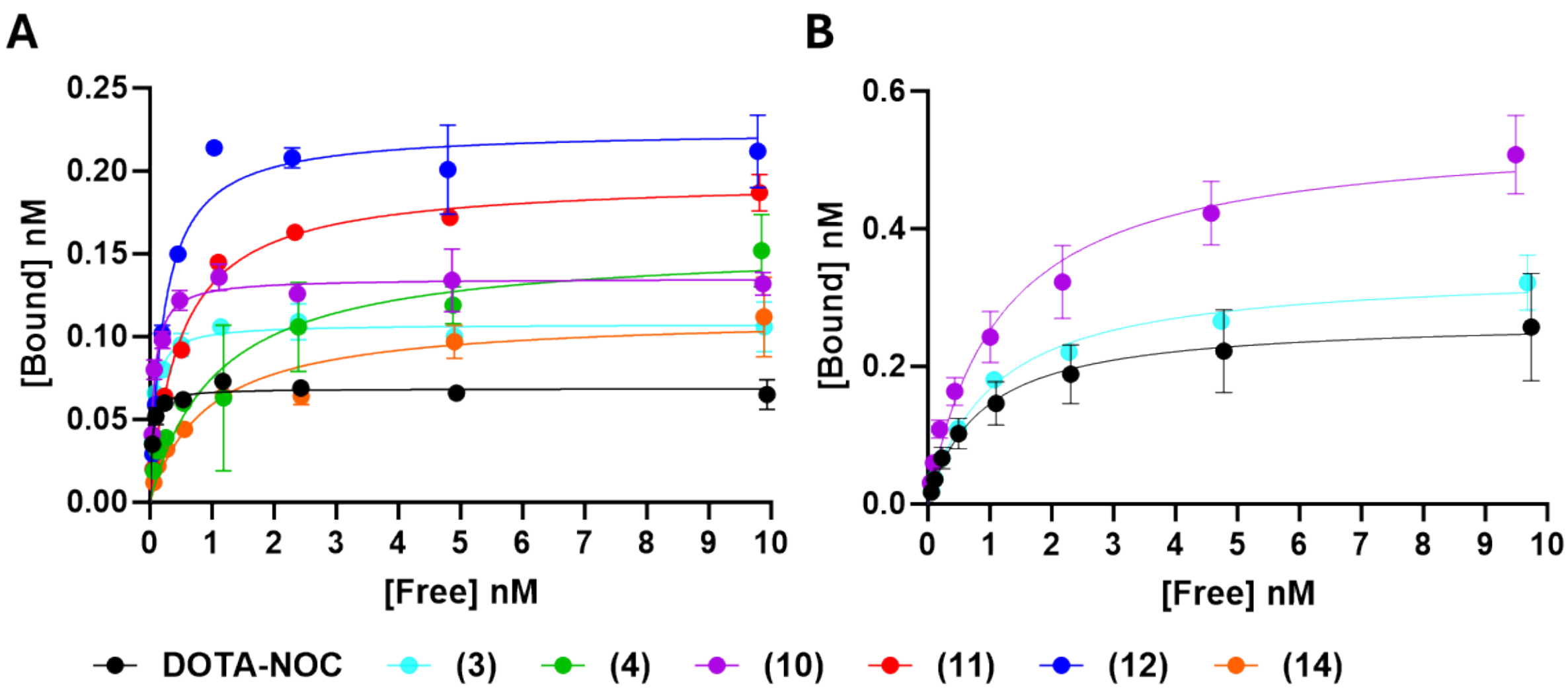

Fig. S3. Saturation binding profile of the [177/nat]Lu-labeled ligands to HEK-SST_2 cell membranes (A) or CHO-SST_5 membranes (B) (10 μg/well). Membranes were incubated with increasing concentrations of each radioligand for 60 min. Data was fitted using the one-phase exponential association equation.

Antagonistic potency

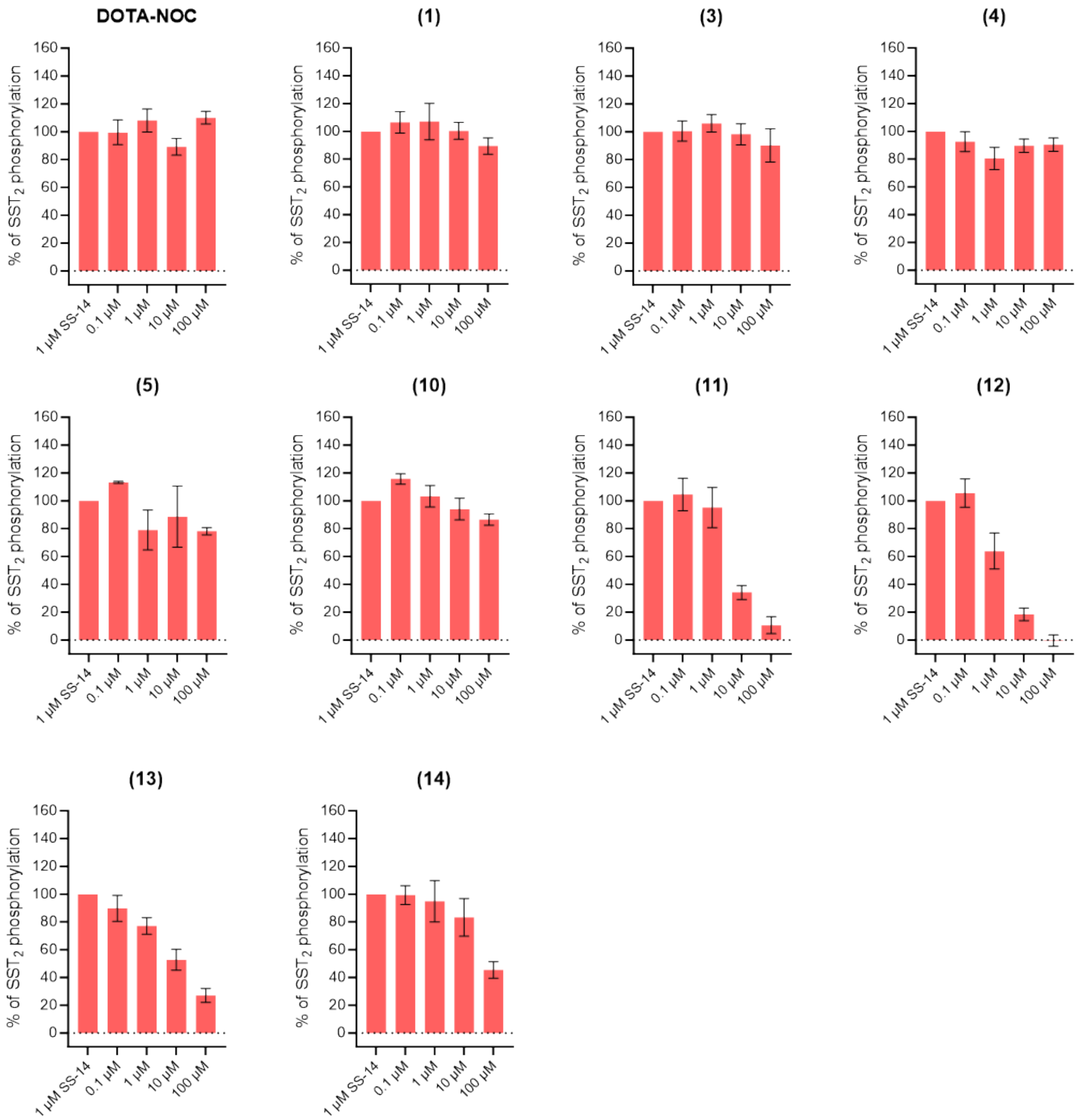

Figure S4. Inhibition of SS-14-induced SST_2 phosphorylation by the tested ligands in a 7TM phosphorylation assay. Phosphorylation of SST_2 at pS341/pS343 was measured following stimulation with 1 µM SS-14 (control, set to 100%). Tested ligands were co-applied at increasing concentrations (0.1, 1, 10, and 100 µM), and their ability to inhibit SS-14-induced phosphorylation was quantified. Data are presented as percentage of SS-14-induced phosphorylation. Bars represent mean ± SD.

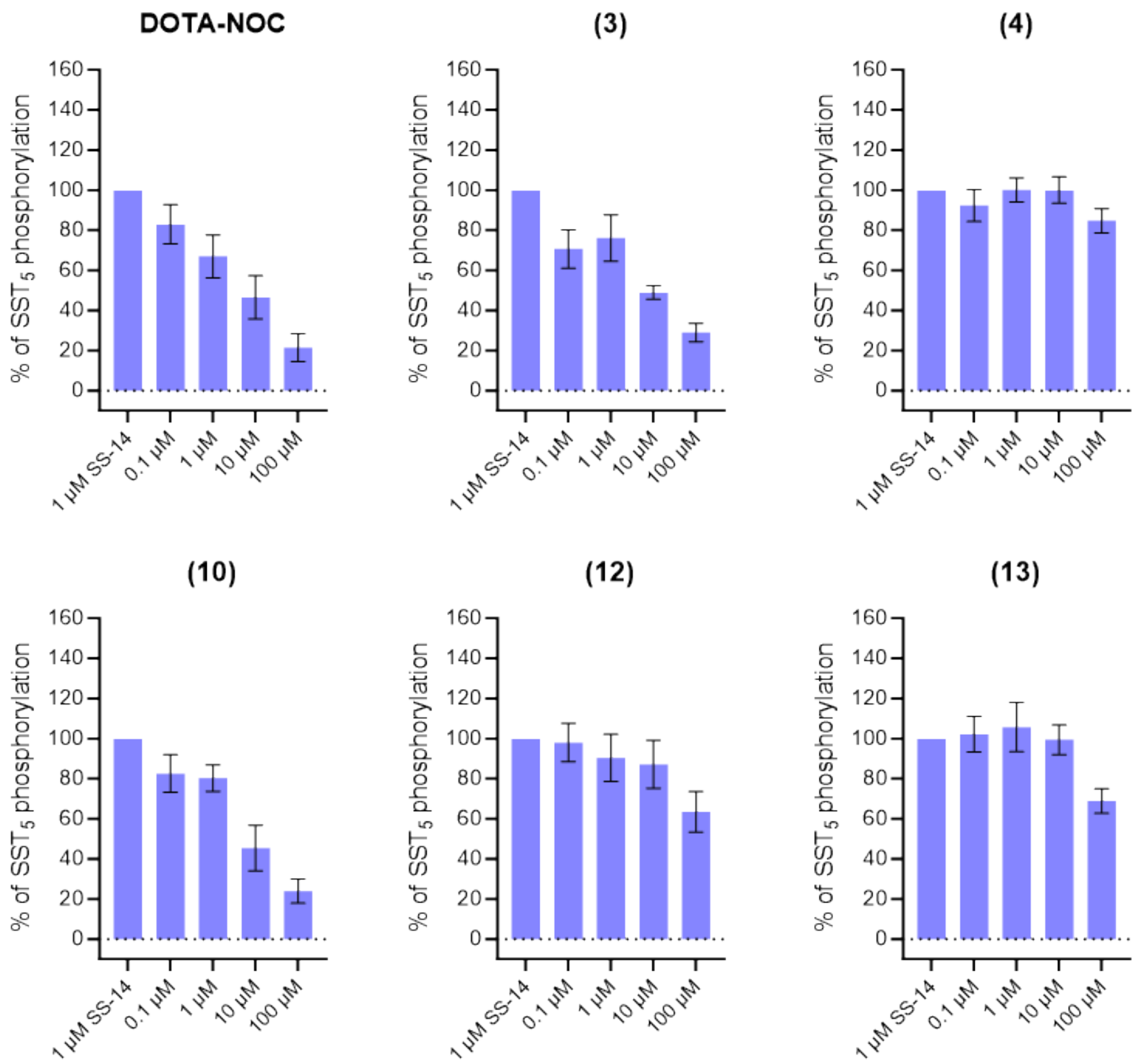

Figure S5. Inhibition of SS-14-induced SST_5 phosphorylation by the tested ligands in a 7TM phosphorylation assay. Phosphorylation of SST_5 at pT333 was measured following stimulation with 1 μM SS-14 (control, set to 100%). Tested ligands were co-applied at increasing concentrations (0.1, 1, 10, and 100 μM), and their ability to inhibit SS-14-induced phosphorylation was quantified. Data are presented as percentage of SS-14-induced phosphorylation. Bars represent mean ± SD.

In vivo specificity of ^{177}Lu-10

Table S5. Biodistribution results of [^{177}Lu]Lu-**10** (10 pmol, 0.4 MBq) in HEK (SST-negative) xenografted nude mice, 1 h p.i.. The results are expressed % IA/g±SD (n=2).

Organ	%IA/g
Blood	1.13±0.32
Heart	1.42±0.28
Lung	15.8±3.37
Liver	2.71±0.59
Pancreas	21.2±2.39
Spleen	1.88±0.19
Stomach	34.8±9.58
Intestine	5.82±1.47
Adrenal	5.12±0.14
Kidney	17.7±4.16
Muscle	0.27±0.06
Femur	1.92±0.53
HEK-tumor	0.84±0.03
